# Field-of-view subsampling: A novel ‘exotic marker’ method for absolute abundances, validated by simulation and microfossil case studies

**DOI:** 10.1371/journal.pone.0320887

**Published:** 2025-05-06

**Authors:** Chris Mays, Marcos Amores, Anthony Mays

**Affiliations:** 1 Geological-Palaeontological Department, Natural History Museum Vienna, Vienna, Austria; 2 School of Biological, Earth and Environmental Sciences, Environmental Research Institute, University College Cork, Cork, Ireland; 3 School of Computer and Mathematical Sciences, University of Adelaide, Adelaide, South Australia, Australia; Maria Curie-Sklodowska University: Uniwersytet Marii Curie-Sklodowskiej, POLAND

## Abstract

Key parameters of biological systems—e.g., productivity, population sizes, biomass—are best expressed as absolute values. Exotic markers (e.g., *Lycopodium* spores introduced into microfossil populations) have long been used to estimate population sizes from representative samples. However, the traditional approach—the ‘linear method’ herein—can be extremely time consuming and impractical for routine use. Here, we present a new variant of this technique: the ‘field-of-view subsampling method’ (FOVS method). This new method requires a few simple, easily obtainable statistical parameters, beyond the standard inputs for the traditional linear method. The FOVS method adds error from sample heterogeneity, but enables the collection of very large sample sizes with low additional effort. We compared the FOVS and linear methods with two case studies: 1, Monte Carlo simulations to validate the methods with idealised datasets; and 2, terrestrial organic microfossils from Permian–Triassic rock strata in southeastern Australia as ‘real-world’ empirical datasets. Three output parameters were measured: 1, absolute abundance; 2, precision (=error rate); and 3, data collection effort (typically, this translates to data collection time). The linear method showed superior efficiency only for assemblages with very low specimen densities and/or near-equivalent target-to-marker ratios, conditions we predict are rare under real-world conditions. In contrast, the FOVS method provided greater precision and/or reduced effort under almost all conditions, without sacrificing accuracy. Although originally developed for microfossils, the new method may apply to any spatial data collection where markers of known quantity can be introduced to a population. Given its demonstrable increased speed and precision, we recommend the FOVS method as the new standard for such absolute abundance estimates. Guidelines and a user-friendly digital interface for implementing both of these count methods are provided, in addition to simulation codes aimed to assist readers in designing their own experiments.

## Introduction

Absolute abundances are key parameters across the biological sciences, including conservation (e.g., species population sizes [1]), biomedicine (e.g., cell counts [2]), agriculture (e.g., herd sizes [3]), and ecology (e.g., biomass [4]; primary productivity [5]). A key strength of absolute abundances is that they are divorced from the issues inherent to proportional (or relative, e.g., percentage) abundance data, such as compositional effects [6–8]. A common compositional effect occurs when a relative increase in one component necessarily results in a decrease of all other components, regardless of whether there is a causal link between them; hence, the analyses of such data can lead to spurious correlations [9]. Moreover, unlike proportional data, absolute values provide standardized metrics for comparing the functioning of organisms, species and ecosystems across spatiotemporal contexts. Since it is generally impractical to count entire populations, statistically significant abundance estimates require an accurate and precise sampling method, but these typically require more data collection effort (or time) than proportional data. A more efficient sampling method will thus enable the accurate measurement of critical biological parameters that were once considered impractical.

When applied to past biological systems, fossil abundance data are used to infer organism spatiotemporal distributions, which can inform myriad environmental, ecological, and evolutionary trends. Relative data can clearly indicate population changes in Earth’s past [10,11], but are most validly applied to limited spatiotemporal contexts (i.e., similar areas and time ranges). However, by providing standardised benchmarks, absolute abundances enable valid comparisons between vastly different contexts, while facilitating additional inferences beyond population shifts. One common absolute abundance metric for past ecosystems—fossil concentration (= number of fossils per unit mass or volume; “c” herein)—quantifies the specific biological contributors to a sediment or sedimentary rock [12]. For several decades, absolute fossil abundances have been applied to uncover diverse aspects of Earth’s past (both Quaternary and pre-Quaternary), including marine (e.g., [13,14]), freshwater productivity (e.g., [15–17]), orbital cyclicity (e.g., [18]), megafauna extinctions (e.g., [19,20]), climate changes (e.g., [21]) and fossil deposition rates (e.g., [22]). Such absolute fossil abundances are particularly important during past, rapid environmental changes. This is owing to the extreme abundance fluctuations of various fossil groups (e.g., algal or ‘acritarch’ blooms [17,23,24]; fungus [25,26] and fern spore abundance spikes [27,28]; charcoal peaks [29,30]). Apparent proliferation events or abundance ‘spikes’ based on relative data might simply reflect the extirpation of other fossil groups. Absolute metrics, on the other hand, can indicate whether group abundances are correlated or independent; this is essential for drawing firm conclusions about the causes of spatiotemporal trends in Earth’s history.

The ‘exotic marker technique’ is a method for measuring absolute abundances. It is performed by simultaneously counting samples (from a given population) of: 1, target specimens (or, specimens of interest); and 2, ‘exotic marker’ specimens (of known total abundance introduced into the population). Crucially, the markers and targets should behave similarly, to result in homogeneous mixing of the two specimen types within the population. As highlighted by Maher [31], this technique has long been utilised in disparate fields, such as agriculture (e.g., counting black sheep among predominantly white flocks) and ecology (e.g., releasing tagged animals into the wild and later counting the ratio of tagged vs non-tagged individuals). First applied to organic microfossils by Benninghoff [32], the exotic marker technique has undergone a series of iterations with different markers [33,34], including distinctive angiosperm pollen (e.g., *Ailanthus altissima* (Mill.) Swingle: [35]; *Alnus incana *subsp. *rugosa* (Du Roi) R.T. Clausen: [36,37]; *Eucalyptus globulus* Labill.: [38–41]; *Nyssa sylvatica* Marshall: [42]), microscopic plastic beads (e.g., [43–46]) or ceramic microspheres [47]. However, the most widely utilised exotic markers are spores of *Lycopodium clavatum* L. [34,48–51], which have been primarily applied to Quaternary sediment samples (e.g., [52–55]) but increasingly to older assemblages (e.g., [14,56,57]). Similar approaches have been successfully applied to other organism groups (e.g., diatoms; [15,16,58]). Whichever exotic marker is used, ideally they should be optically distinct from the indigenous specimens, whilst having similar behavioural properties (e.g., the same hydrodynamic and chemical characteristics as the microfossil targets) to avoid overrepresentation of either group [59].

This study aims to statistically assess and refine the ‘exotic marker technique’, a commonly utilised measure of absolute abundances. Two parallel data sets—computer-based simulation and organic microfossil assemblages—will be collected to test a new variant of this technique (the ‘field-of-view subsampling method’, or FOVS method), and compare it to the traditional method (the ‘linear method’ herein). The FOVS method has the potential to provide greater efficiency (improved precision and/or reduced data collection effort) and increase the versatility of legacy samples or previously collected spatial data. Further, we aim to maximise the utility of the count methods by providing: 1, precise criteria for choosing the optimal method for each sample; 2, the source code for the simulated data sets; and 3, an accessible user interface for estimating key parameters and employing both methods.

## Materials and methods

### Specimen absolute abundances

The exotic marker technique measures the absolute abundances of the specimens of interest, or ‘targets’. A common absolute abundance is specimen concentration (c), measured as the number of targets per unit size (e.g., mass, area or volume). For concentration estimates, one must: 1, measure the original sample size; 2, add exotic markers of known abundance and uncertainty (e.g., standard deviation); and 3, compare the counted abundances of both targets and exotic markers from a sample. Since we know the number of introduced marker specimens per unit size (i.e., exotic marker concentration) *a priori*, we can infer the concentration of indigenous specimens by measuring the ratio of targets to markers in a sample of that population. In this fashion, exotic markers enable estimates of target concentrations in their original, natural populations, even when the samples have been removed from those populations (e.g., for cell or microorganism counts).

The general formula for these concentrations (c) follows Benninghoff [32], with terms updated from [31]:


$$\begin{array}{c} c=\frac{x\times N_{1}\times {\overset{―}{Y}}_{1}}{n\times \overset{―}{V}} \end{array}$$ (1)

where x = the total target (e.g., microfossil) specimens counted, $N_{1}$ = the number of doses (e.g., tablets) of exotic markers added to the sample, ${\overset{―}{Y}}_{1}$ = mean number of exotic markers per dose, n = exotic marker (e.g., *Lycopodium* spore) specimens counted, and $\overset{―}{V}$ = total size (mass, area or volume) of sample. Unless specified, c indicates concentrations derived using the originally proposed concentration method, dubbed the ‘linear method’ herein (see ‘linear method: operation’ below). The term for linear method concentrations may also take the form $c_{L}$ herein to differentiate it from $c_{F}$, the latter of which is the concentration value from the newly proposed ‘FOVS method’.

Significance tests were conducted with PAST v. 4.13 [60] to statistically compare the outputs of the two methods. A glossary of terms and abbreviations used in this study is provided in S1 Table.

### Counting techniques

Here, we outline the rationale, key parameters and practical aspects of the two count methods for absolute abundances: 1, the linear method; and 2, the field-of-view subsampling (FOVS) method.

**Fig 1:**
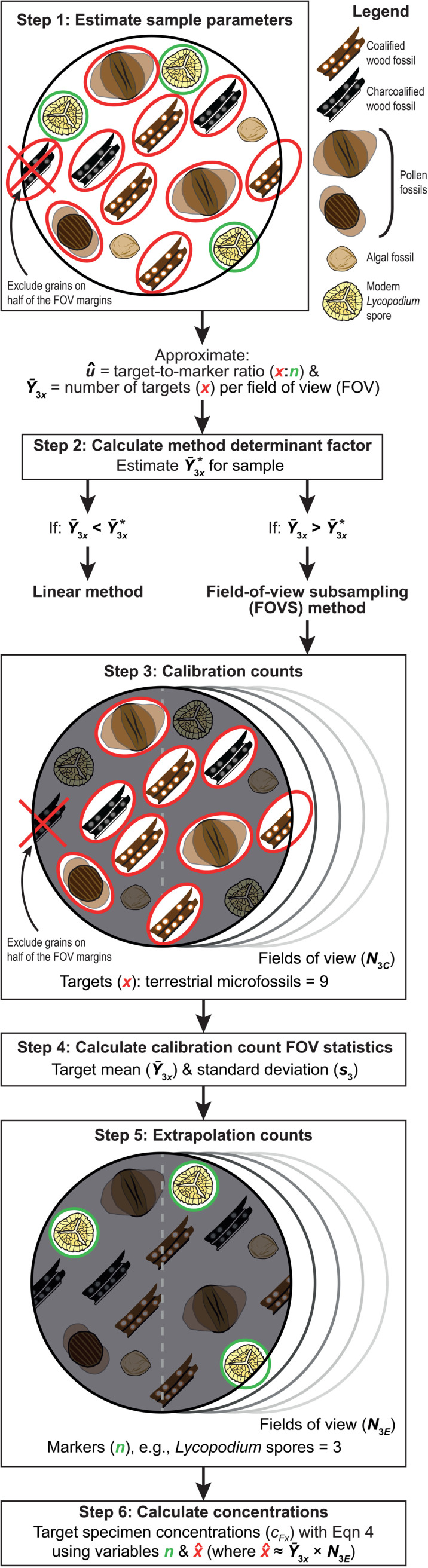
A flowchart of two count methods for estimating absolute abundances using exotic markers, with an organic microfossil case study. The flowchart includes a step-by-step procedure of the field-of-view subsampling (FOVS) method. To calculate ${\overset{―}{Y}}_{3x}^{*}$ for determining the most efficient method for each sample, use Eqn 20 when x>n; if x<n, we recommend calculating ${\overset{―}{Y}}_{3n}^{*}$ instead with S11 Eqn (see S1 File for more details). Note: an additional input parameter for Eqn 20 is the researcher-specific time parameter ω (= ratio of time taken for field-of-view transitions vs individual specimen counts). FOV = field of view; for full descriptions of mathematical terms, see S2 File. A user-friendly interface for all calculations is available here.

The method determination process is summarised in steps 1 and 2 of Fig 1, but is discussed in detail later (see the ‘choosing the superior count method’ section) since this requires knowledge of the key sample and count parameters discussed in the immediately following sections. The sections preceding ‘choosing the superior count method’ assume that the choice of method has already been made.

#### Linear method: Operation.

The ‘linear method’ (Eqn 1) has been commonly used to estimate specimen abundances in a population, whereby concentrations of thousands or even millions of specimens (per unit mass or volume) can be estimated from only a few hundred. This method involves the identification and counting of individual specimens, and their assignment to two (or more) specimen categories: markers (n) and targets (x or, for multiple target categories, $x_{1},x_{2},\ldots \;x_{k}$).

Following [31], this procedure can be illustrated by an agricultural analogy. A farmer introduces 1000 black sheep into a large flock of white sheep of indeterminate size. After a modest interval, during which the black and white sheep subpopulations mingle (assuming the sheep do not discriminate based on colour), the farmer herds the sheep into a line. The farmer then counts a sample of sheep until the number of white sheep (the targets, or x) reaches 100. Assume that among this random sampling, the farmer also counted ten black sheep (the markers, or n). By extrapolating the white-to-black sheep ratio (10:1) to the whole flock, the farmer estimates the total white sheep population as 10,000.

This can be considered a linear procedure, whereby specimen counts (both x and n) are collected simultaneously and sequentially. In the simplest case, this is conducted until a pre-determined minimum value of x is reached (although one could also use pre-determined n values). Stopping when a predetermined number of target specimens is reached defines a random “window” of a sample area (e.g., microscope slide) within which we now have a random number of markers. In the microfossil case study, the target population is the total number of microfossils in a given sediment or sedimentary rock, and the marker population is the total number of introduced exotic markers (e.g., *Lycopodium* spores). Since all specimens in a sampled area are examined individually and assigned to a count category, the linear method tends to provide accurate target identifications.

The highest precisions for concentration estimates are achieved when the target specimens and exotic markers are equal in the population (i.e., x=n; Regal & Cushing [61]). We recover this result as a by-product of mathematical analyses comparing the efficiency of the two methods (see S1 File). However, for combined (x+n) specimen counts of several hundred (e.g., ≥ 500), a ‘target-to-marker ratio’ (or $\hat{u}$, following the terminology of [31]) close to 2:1 provides the most efficiency by striking the optimum balance between precision and data collection effort [31]. For this target-to-marker ratio ($\hat{u}\approx 2$), count sizes greater than 500 provide sharply diminishing returns with increased effort (translating to increased data collection time). Similarly, Price et al. [51] recommended that the target-to-marker ratio should be < 5:1 (or $\hat{u}<5$). In cases where the target and marker ratios are near-equivalent (for single target counts), precise concentrations can typically be achieved with target counts of less than 300 [49].

(Note: unless stated otherwise, we assume that the targets are more common than the exotic markers; hence, we utilise the term ‘target-to-marker ratio’ in place of ‘common-to-rare ratio’. If, however, the markers are more common than the targets, see the relevant section in the S1 File.)

#### Linear method: Precision estimates.

To gauge the precision of concentration estimates using the linear method, concentration total error values ($\sigma_{L}$) were calculated with the formula (from [34], updated with terms defined by [31]):


$$\begin{array}{c} \sigma_{\text{L}}=100\sqrt{{\left(\frac{s_{1P}}{\sqrt{N_{1}}}\right)}^{2}+{\left(\frac{\sqrt{x}}{x}\right)}^{2}+{\left(\frac{\sqrt{n}}{n}\right)}^{2}} \end{array}$$ (2)

where $\sigma_{L}$ is the total standard error of the linear method concentration estimate (in %). In this formula, the variables x, n and $N_{1}$ are as in Eqn 1, while $s_{1P}$ is the proportional sample standard deviation of the number of exotic markers per dose (see S1 Table).

The standard deviations ($\sqrt{x}$ and $\sqrt{n}$) come from the underlying assumption that all specimens (targets and markers) are independently distributed according to a Poisson point process (i.e., they are distributed uniformly at random over the study area and, hence, their distributions are independent of each other). These assumptions provide a valid statistical framework for many data in the biological [62] and physical [63] sciences. The standard deviations are then divided by the respective number of counted specimens to yield proportional standard deviations. Note that since the number of targets (x) (or, in some cases, the number of markers [n]) is pre-determined in the linear method, the inclusion of error terms for both may seem counterintuitive. However, the error term coming from the fixed quantity can be understood as the contribution to the error from the variable size of the linear method window determined by the random locations of the counted specimens.

Since the specimens are counted sequentially, neither density nor homogeneity (defined here as the variance of the specimen density across the study area) will impact the precision of this method. However, these factors will certainly affect the ‘data collection effort’, which is reflected by the time spent during counting (e.g., at the microscope, sparsely populated slides will take a longer time to achieve a given count size). We have not provided a comprehensive discussion of the effects of inhomogeneity, since we do not yet have quantitative data to test this; however, this would serve as the basis for future investigation.

Precision of linear method concentration was also estimated by calculating confidence intervals for $c_{L}$. For these, the parametric approach by Maher (1981, p. 179) was implemented, the relevant parameters and formulae for which are summarised in S1 Table. A minor change to the confidence interval calculation was made to adapt it to sample mass (rather than volume), following [49]. Maher’s [31] calculations encompass the uncertainty in size (in units of volume or mass) and number of samples. In the microfossil case study below, the masses were considered true values, and standard errors were set to 0.1 g (equivalent to the precision limits of the instrument used for measuring mass).

#### Field-of-view subsampling (FOVS) method: Operation.

The FOVS method is a count data collection approach that we have designed as an alternative, or complement, to the linear method. This area-based sampling is distinct from the linear method above, for which both target (x) and marker (n) specimens are counted in a continuous sequence. Instead of linear, stepwise counts of all individual specimens, the FOVS method obtains large abundance data sets by extrapolating abundances from surface area subsamples.

To illustrate the differences between the linear and FOVS methods, let’s consider a modified version of the agricultural analogy described above (see ‘linear method: operation’). As before, the farmer introduces a known number of black sheep into a white sheep flock of larger but indeterminate size. This time, however, the farmer adopts a novel perspective: examining the flock from satellite. Even at this scale, the black sheep are clearly distinct from the white sheep. Using a consistent field-of-view size (e.g., 100 m^2^), the farmer first establishes the mean and standard deviation of white sheep (the targets, or x) from a subsample of fields of view across the meadow. This ‘calibration count’ is followed by a second count (the ‘extrapolation count’), whereby another independent subsample of fields of view from the same meadow is collected, but this time counting only the distinctive and less common black sheep (the markers, or n). Given the relative rarity of black sheep, the farmer can quickly scan a large quantity of fields of view, building a large sample size of black sheep markers during this second stage. The farmer can then infer an enormous sample size of white sheep targets by combining the mean number of white sheep per field of view in the first count with the ratio of black-to-white sheep provided by the second count.

Area-based subsampling techniques have been applied widely across disparate sciences to infer population statistics [64], but—to our knowledge—never before to organic microfossil assemblages (cf. [16]). For example, quadrat sampling is a standard method used in field ecology, whereby the specimens within a set of representative areas are counted, from which populations can be extrapolated across a broader study area [65,66]. We propose that the microscope field of view (FOV) can be utilised as the unit of surface area, in place of the quadrat. For valid results, the areas of the subsamples or ‘fields of view’ should be consistent for all counts of a given assemblage.

The essential difference between standard quadrat methods and the FOVS method proposed herein is the addition of exotic markers of known quantity (Eqn 1). This is because quadrats cannot be applied directly to samples of target specimens (e.g., cells, microorganisms) that have been isolated from their original populations (e.g., a patient, a body of water or a sedimentary stratum). This process of isolation results in specimen distributions and concentrations that are very different to those in the natural systems from which they were sampled. In such isolated contexts, the observed proportions of targets to markers provide an accurate assessment of total target concentrations within a population from which a sample has been taken. If the additional uncertainty derived from the field-of-view statistics is offset by the larger sample sizes, this combination of concentration estimation and surface area subsampling holds the potential for precise absolute abundances with minimal sampling effort.

The FOVS method involves the following sequence of steps, which are summarised in Fig 1; see S1 Table for equations and terms. (Note: The following procedure assumes that the FOVS method has been determined as the most appropriate for the given population [Fig 1, steps 1, 2]. For more details on the method determination process, see discussion in ‘choosing the superior count method’).

**Fig 2:**
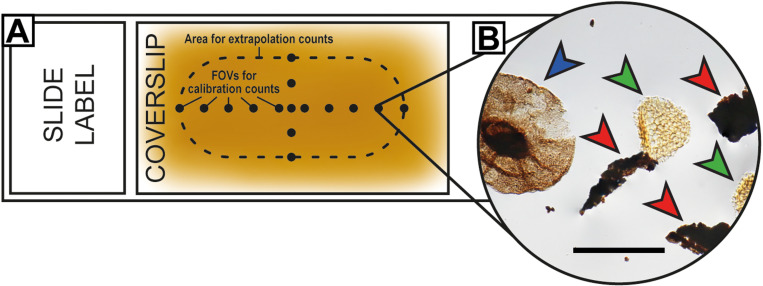
Field-of-view subsampling (FOVS) method demonstrated with a schematic microscope slide of organic microfossils. (A) Organic microfossil slide, with specimens dispersed across a coverslip. For valid subsampling: 1, the spatial range of the extrapolation-count fields of view (FOVs) should match those of the calibration counts; 2, calibration counts should be conducted with representative fields of view (e.g., both x- and y-axes, or randomly distributed); and 3, prevent edge effects by avoiding counts near the coverslip margins. (B) A typical field of view of an organic microfossil (palynological) slide with potential targets and markers; red and blue arrows: terrestrial microfossils, fossil wood fragments (red) and a fossil plant spore, *Playfordiaspora crenulata* (blue); green arrows: exotic markers (modern *Lycopodium clavatum* spores); distinctive optical features of the markers are necessary for accurate and rapid data collection using the FOVS method; scale = 50 μm (sample S090320, Bonneys Plain-1, 301.56 m).


A. Examine the assemblage to determine whether the targets or markers are more common. (For the following example, the target specimens, x, are assumed to be more common. If markers, n, are more common than x, see the S1 File.)
B. Conduct a series of ‘calibration counts’ for the target specimen type (Fig 1, step 3). These consist of counts of all target specimens in a subsample of fields of view (where $N_{3C}$ is the total number of calibration-count fields of view). To avoid overrepresenting the number of target specimens per unit area, exclude half of the specimens on the field-of-view margins (e.g., on one side of the field of view; cf. [67]).
C. Calculate the mean (${\overset{―}{Y}}_{3}$) and standard deviation ($s_{3}$) of the target specimens per field of view from the calibration counts (Fig 1, step 4).
D. Conduct an ‘extrapolation count’ of new fields of view (Fig 1, step 5), whereby two values are collected simultaneously: 1, the number of extrapolation-count fields of view ($N_{3E}$); and 2, the number of rare specimens (typically, the exotic markers, n). To ensure that the calibration counts are representative, the regions chosen for both the calibration- and extrapolation-count fields of view should be located close to each other on the microscope slide. In this way, the specimen density and heterogeneity will be approximately equivalent (Fig 2A).
E. Organic microfossil concentration can then be calculated (Fig 1, step 6). Assuming the target specimens were the common specimens during the calibration counts, then an approximate value for x can be calculated by multiplying the mean number of targets per field of view from the calibration counts (${\overset{―}{Y}}_{3x}$) by the counted number of fields of view from the extrapolation counts ($N_{3E}$). This extrapolated value of x for the extrapolation count is denoted $\hat{x}$, i.e.,

$$\begin{array}{c} \hat{x}={\overset{―}{Y}}_{3x}\times N_{3E}. \end{array}$$ (3)

The subscript x in the term ${\overset{―}{Y}}_{3x}$ indicates that the target specimens are the most common specimen type, and subjects of the calibration counts. The total number of exotic markers from the extrapolation counts (n) can then be inserted directly into a modified version of Eqn 1:


$$\begin{array}{c} c_{Fx}=\frac{\hat{x}\times N_{1}\times {\overset{―}{Y}}_{1}}{n\times \overset{―}{V}}, \end{array}$$ (4)

where we denote the estimated organic microfossil concentration from the FOVS method by $c_{Fx}$ to distinguish it from the linear method concentration estimate ($c_{L}$; see Eqn 1). (The subscript “x” for $c_{Fx}$ denotes that the target specimens are the foci of the calibration counts; if this is not the case, see the S1 File).

#### Field-of-view subsampling (FOVS) method: Precision estimates.

While both absolute abundance methods (linear and FOVS) estimate the population values from subsamples, the accuracy of common specimen abundances using the FOVS method depends on the representativeness of the fields-of-views during the calibration counts. This entails a different source of error compared to the linear method. As such, the calculation of total error in Eqn 2 needs to be modified as follows:


$$\begin{array}{c} \sigma_{\text{F}x}=100\sqrt{{\left(\frac{s_{1P}}{\sqrt{N_{1}}}\right)}^{2}+{\left(\frac{s_{3P}}{\sqrt{N_{3C}}}\right)}^{2}+{\left(\frac{\sqrt{n}}{n}\right)}^{2}} \end{array}$$ (5)

where $\sigma_{Fx}$ is the total standard error of the concentration mean for the FOVS method (in %). In this function, $s_{1P}$ and $N_{1}$ are as in Eqn 2, while $s_{3P}$ is the proportional sample standard deviation for the target specimens from the calibration counts and $N_{3C}$ is the number of fields of view during the calibration counts. For further calculations and descriptions of terms, see S1 Table.

Note that we have scaled $s_{3P}$ by the so-called $c_{4}\;$correction, which is needed to ensure an unbiased estimation of the standard error of a sample mean with an underlying normal distribution (without this correction, the standard error is expected to understate the true value). This appears to be a classical result, with the earliest unambiguous reference we can find being [68] (eqn 16 therein). This $c_{4}$ correction is an artefact of the square root taken to obtain a standard deviation, and so if one uses the variance then it is not needed. However, standard deviation is more commonly cited in the literature, and so we utilise the more widely used standard deviation formulation in Eqn 5, but include the conservative correction in the $s_{3P}$ term (see the S1 File).

**Fig 3:**
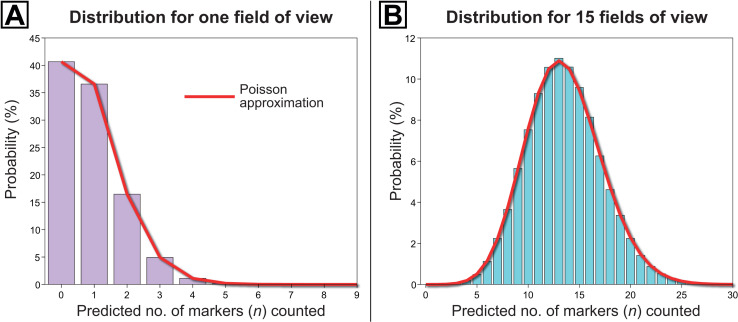
Histograms of counted marker specimens for an assemblage with rare markers; in this case, target-to-marker ratio [$\overset{―}{\overset{―}{u}}$] = 30; expected number of rare grains per field of view (${\overset{―}{Y}}_{3n}$) = 0.9; the number of simulations was 100,000. These histograms are compared to the Poisson probability mass function (red curves) with rate given by the actual mean number of markers per field of view calculated from the simulations. (A) Histogram for a single random field of view. (B) Histogram for the sum of 15 random fields of view.

Owing to their large sample sizes, standard sampling theory [64] tells us that the abundances of marker specimens per dose and the mean number of common specimens in the calibration-count fields of view ($N_{3C}$) both approximate Gaussian (or ‘normal’) distributions. Hence, their error functions—represented by the relationships between terms $s_{1P}$ and $N_{1}$, and $s_{3P}$ and $N_{3C}$, respectively—take similar forms in Eqn 5. This differs from the uncertainty associated with the target specimens (x) in Stockmarr’s [34] total error estimate for the linear method (Eqn 2), where a single measurement for x is taken and the statistics are assumed to have a Poisson distribution. However, given the typically low absolute abundances of the rare specimen (n) counts per field of view (often between 0.2 and 5), the uncertainty estimate associated with n still likely follows a Poisson distribution. This type of distribution is particularly appropriate for discrete count data that are very rare (e.g., have a relatively low probability of occurrence within a given field of view; [69]). To demonstrate this, we present simulated data sets of rare specimen counts (mean number of rare specimens per field of view: 0.9, iterations: 100,000), and compare the probability distributions of two counts (Fig 3): 1, a single field of view ($N_{3C}=1$); and 2, the sum of 15 fields of view ($N_{3C}=15$). The red curves are the Poisson approximations, showing a very good visual fit, and the sum of squared residuals of 1x10^-7^ for the fit to the single field-of-view histogram and 1x10^-5^ for the total of 15 calibration-count fields of view. This allows us to conclude that the Poisson error estimate $\frac{\sqrt{n}}{n}$ for the rare grain type is a suitable approximation.

As in the case of the linear method, multiple target specimen types may be assigned for the FOVS method (e.g., $x_{1},x_{2},\ldots ,\;x_{k}$). For the FOVS method; however, the means per field of view (${\overset{―}{Y}}_{3,1},{\overset{―}{Y}}_{3,2},\;\ldots ,\;{\overset{―}{Y}}_{3,k}$) and standard deviations ($s_{3,1},\;s_{3,2},\;\ldots ,\;s_{3,k}$) of each target will need to be collected during the calibration counts. Combined with the markers (n) from the extrapolation counts, and following the sequence of steps outlined above (see ‘field-of-view subsampling (FOVS) method: operation’), their individual concentration estimates could then be separately derived using Eqn 4. The number of appropriate targets is dependent on the relative abundances of these targets in the assemblage (i.e., the evenness of the assemblage). Should these additional targets be particularly rare, then their calibration counts will likely result in large standard deviations; hence, their predicted total error estimates will be correspondingly large.

It is predicted that for routine data collection effort (e.g., total counts of several hundred) the FOVS method will provide superior concentration precision (lower total error values) to the linear method for assemblages with suboptimal target-to-marker ratios (e.g., $\hat{u}>3$). This is because in cases with disproportionately abundant common specimens, there will be diminishing returns in their additional counts [31], at which point, the key limiting factor in concentration precision will be the number of rare specimens. The power of the FOVS method lies in its ability to detect greater absolute abundances of rare specimens with minimal additional sampling effort, thus mitigating this issue. However, if the target relative abundance is exceedingly high—resulting in a very large target-to-marker ratio (e.g., $\hat{u}>100)$—then it is predicted that not even the FOVS method will salvage satisfactorily precise concentrations without impractical sampling efforts, as the precision still relies on the absolute number of exotic markers counted. We test these predictions below by comparing the efficiencies of the FOVS and linear methods.

### Estimating data collection effort

Given that the FOVS and linear method counts are conducted in subtly different ways, the amount of collection effort is not directly comparable. To standardise these comparisons, time was utilized as a proxy for data collection effort for both methods. The accurate identification and counting of target and marker specimens takes data collection effort (=time). Herein, we assumed an equivalent time required for the identification and counting of targets and markers. This assumption is most valid for analysts working at ‘low taxonomic resolution’ (broad, easily identifiable categories, e.g., terrestrial organic microfossils) or low diversity assemblages, where the targets can be rapidly identified and distinguished from the markers. However, for the purposes of our comparisons of the linear and FOVS count methods, any differences in identification time in each assemblage would be consistent for both methods, thus mitigating their effects on the validity of the comparisons (see the end of this section for a brief discussion of potential effort asymmetry between targets and markers). Moreover, the effort required for the calibration counts of the FOVS method is analogous to the counts in the linear method (for a given assemblage), since both require: 1, the same microscopy conditions (lighting, magnification, etc.); and 2, the identification and counting of specimens on a given field-of-view before transitioning. In terms of effort, the primary difference is the addition of the subsequent ‘extrapolation counts’ for the FOVS method. These extrapolation counts typically entail a greater number of field-of-view transitions (i.e., the movements of the microscope stage to new fields-of-view), but fewer individual specimen identifications.

Sample collection effort is also a function of specimen density, which can vary greatly between assemblages. For example, the number of specimens on a given slide may be homogeneous but sparse, which would necessitate a larger number of field-of-view transitions, hence, a longer data collection time. Lower magnification fields of view would minimise the number of transitions, but are likely to decrease identification accuracy. The similarity between the FOVS method calibration counts and the linear method means that these two processes will be affected by specimen density to a similar degree. But the typically higher number of field-of-view transitions in the FOVS method means that this can represent a major component of the method’s data collection effort, especially for low density assemblages.

For these reasons, a fair comparison requires that the numbers of examined fields of view be factored into the effort estimates of both methods. While field-of-view transitions may be extremely rapid (e.g., a few seconds on a given microscope slide), it may be generally slower than specimen counts within a single field of view. Microscopy trials counting total terrestrial organic microfossils yielded a time value for each field-of-view transition as approximately twice that of counting a single specimen on one field-of-view. However, we recognise that field-of-view transition effort factor (here denoted ω, which is equal to the quotient of mean field-of-view transition time and mean specimen count time) and the identification of targets or markers may vary greatly between researchers and the types of targets being sought. For example, differentiating between visually similar targets to ensure accurate identification may inflate the specimen count time.

With all this in mind, the data collection effort for the linear method ($e_{L}$) was calculated as follows:


$$\begin{array}{c} e_{L}=\left(\omega \times \frac{x}{{\overset{―}{Y}}_{3x}}\right)+x+n, \end{array}$$ (6)

where ω is the field-of-view transition effort factor, x is the counted number of target specimens, n is the counted number of marker specimens and ${\overset{―}{Y}}_{3x}$ is the mean number of targets per field of view. For the microfossil case study herein, ${\overset{―}{Y}}_{3x}$ was collected during the FOVS method calibration counts from the same slides of each sample. However, if linear method effort were to be estimated without this, the number of fields of view would need to be counted and substituted for the term $\frac{x}{{\overset{―}{Y}}_{3x}}$ in Eqn 6, analogous to $N_{3C}$ for the FOVS method (see below).

In contrast, data collection effort for the FOVS method ($e_{F}$) was calculated by the formula:


$$\begin{array}{c} e_{F}=(\omega \times N_{3C})+x+(\omega \times N_{3E})+n. \end{array}$$ (7)

In this formulation, the sum of the common (typically, target) specimen counts (x) and the calibration-count fields of view ($N_{3C}$) collectively represent the effort during the calibration counts. The effort of the extrapolation counts is represented by the sum of the last two terms: the number of extrapolation-count fields of view ($N_{3E}$) and the number of rare (typically marker) specimens (n).

As noted above, the target (x) and marker (n) specimen identifications entail equivalent sampling effort (=time) in the effort equations (Eqns 6 and 7). These equations are generally valid for the empirical application outlined in the present paper (e.g., terrestrial organic microfossil) because many categories of target are easily discernible from other specimens, including the markers. In such cases of low taxonomic resolution, there is low target identification effort and a negligible difference in effort between targets and markers. However, these versions of the effort equations are invalid when targets require higher degrees of scrutiny to verify their identities; for example, when differentiating between morphologically similar categories (‘high taxonomic resolution’). Targets may require relatively high effort compared to the markers. In such cases, an ‘effort asymmetry factor’ should be applied to x. For example, a factor of 5 applied to x would indicate that targets take five times longer to identify than markers. Our preliminary experiments inserting various values of this asymmetry factor into Eqns 6 and 7 show that linear method effort increases disproportionately with increasing count asymmetry (i.e., with a relative increase in target identification effort). This can be understood in the following way: the linear method requires detailed scrutiny of all targets throughout the entire data collection process, while this same level of scrutiny is only needed during the calibration counts of the FOVS method (assuming targets are more common, and the subject of the calibration count). Hence, it is highly likely that the FOVS method will prove more efficient than the linear method for studies that require high taxonomic resolution. However, the full implications of this effort asymmetry have not yet been explored and are worthy of a future systematic analysis.

### Simulating precision as a function of data collection effort

The simulations enable us to define the assemblage characteristics precisely, against which we can test the efficiency of each method by how close these methods reach the predefined values. Hence, in the simulated case study, we can denote these pre-defined ‘true’ values of targets ($\overset{―}{\overset{―}{x}}$) and markers ($\overset{―}{\overset{―}{n}}$), with the population ratio of target to marker specimens ($\overset{―}{\overset{―}{u}}$) as


$$\begin{array}{c} \overset{―}{\overset{―}{u}}=\frac{\overset{―}{\overset{―}{x}}}{\overset{―}{\overset{―}{n}}\;}\geq \;1, \end{array}$$ (8)

which reflects the typical scenario whereby the target specimens are more common than the exotic markers (following Eqn 3). While $\overset{―}{\overset{―}{u}}$ is known in the simulations, the sample target-to-marker ratio ($\hat{u}$) is an approximation of this value; this sample variable is given by: 1, $\hat{u}=x/n$ for the linear method; or 2, $\hat{u}={\overset{―}{Y}}_{3x}/{\overset{―}{Y}}_{3n}$ for the FOVS method, where ${\overset{―}{Y}}_{3x}$ is the mean number of targets per field of view, and ${\overset{―}{Y}}_{3n}$ is the mean number of markers per field of view.

#### Linear method.

For the linear method, we note that the expected amount of effort is directly proportional to the number of specimens that we count in a window. To see this, we substitute $n=\hat{u}\times x$ into Eqn 6 to measure linear method effort, thus obtaining


$$\begin{array}{c} e_{L}=\left(\frac{\omega }{{\overset{―}{Y}}_{3x}}+1+\frac{1}{\hat{u}}\right)x=Ax, \end{array}$$ (9)

We can then substitute A (which reflects the degree of effort for each specimen in the linear method) into Eqn 2 to give us an expression for the precision as a function of the amount of effort:


$$\begin{array}{c} \sigma_{L}(e_{L})=100\sqrt{\;T+\left(1+\hat{u}\right)\frac{A}{e_{L}}}, \end{array}$$ (10)

where T is the error contribution from the marker doses ($T={s_{1P}}^{2}\text{/}N_{1}$; e.g., *Lycopodium* spore tablets). As we increase the data collection effort ($e_{L}$), we naturally expect an increase in precision (i.e., a decrease in error, $\sigma_{L}$). Our derivations of Eqn 10 (see the ‘analysis of precision as a function of effort and the ratio of common and rare grains’ section of S1 File) reveal: 1, there are steeply diminishing returns from increased effort with the linear method, as error decreases with the square of the effort (see S1 Eqn); and 2, that the highest precision estimates of the concentration are achieved when $\hat{u}=1$ (i.e., x=n; S2 Eqn), thus confirming the result by Regal & Cushing [61] mentioned in the introduction.

#### FOVS method.

We would like to obtain an estimate of the improvement in FOVS method precision ($\sigma_{F}$) for increased effort ($e_{F}$), as we did for the linear method. However, we cannot do this directly, since the FOVS method involves two independent variables that determine the amount of effort: 1, the number of calibration fields of view ($N_{3C}$), which increases x; and 2, the number of extrapolation-count fields of view ($N_{3E}$), which increases n. Yet, if we can find the optimal ratio of calibration- and extrapolation-count fields of view, then we will reduce our two-dimensional problem to a single dimension. Ultimately, this was done by employing a standard optimisation technique: calculate the derivative of the FOVS method error function (Eqn 5) and set it equal to zero (see ‘FOVS method optimisation’ below).

To make this derivative tractable, however, we will assume that the error term for the common specimens ${\left(\frac{s_{3P}}{\sqrt{N_{3C}}}\right)}^{2}$, which will typically be the target specimens, is well-approximated by the Poisson result ${\left(\frac{x}{\sqrt{x}}\right)}^{2}$. Given that this approximation is quite good for small numbers (as shown in Fig 3), we expect that this Poisson assumption will not introduce any error into our determination of the optimal field-of-view count ratio, since any small deviations will be erased when the numbers of calibration-count and extrapolation-count fields of view are rounded off to the nearest integer. The assumption may introduce a small degree of error into the formula for choosing between the FOVS and linear methods (see ‘choosing the superior count method’); however, our predictions for this choice match the data generated in the simulations (see S1 File). Thus, we have confidence that the assumption is sound for this purpose, especially given the large differences in $\hat{u}$ values expected in real assemblages.

So, with the assumption of a Poisson distribution for the target grain error term, we make the replacement $x=N_{3C}{\overset{―}{Y}}_{3x}$, using the definition of ${\overset{―}{Y}}_{3x}$ in Eqn 3. (Note: x is the number of targets counted during the calibration counts.) Also, using the estimate for the average density of marker grains in each extrapolation-count field of view ${\overset{―}{Y}}_{3n}={\overset{―}{Y}}_{3x}/\hat{u}$, we make the following replacement: $n=N_{3E}{\overset{―}{Y}}_{3n}=\frac{N_{3E}{\overset{―}{Y}}_{3x}}{\hat{u}}$. Substituting these into Eqn 5 gives us


$$\begin{array}{c} \sigma_{F}\left(N_{3C},N_{3E}\right)=100\sqrt{T+\frac{1}{N_{3C}{\overset{―}{Y}}_{3x}}+\frac{\hat{u}}{N_{3F}{\overset{―}{Y}}_{3x}},} \end{array}$$ (11)

where we have included the arguments of the function to explicitly denote that this is a two-dimensional function, and to make the following calculations clearer.

From the results of the derivations (see S1 File for details of this procedure), the increase in precision decreases with the square of the effort (S3 and S4 Eqns). This is analogous to the linear method (S1 Eqn) and, once again, the error depends in a non-trivial way on the target specimen density (${\overset{―}{Y}}_{3x}$) and the target-to-marker ratio ($\hat{u}$).

***FOVS method optimisation*:** The FOVS method error depends in large part on the numbers of both calibration-count fields of view ($N_{3C}$) and extrapolation-count fields of view ($N_{3E}$). To find the optimal ratio of these counts, first we solve Eqn 7 for $N_{3E}$


$$\begin{array}{c} N_{3E}=\frac{e_{F}-\left(\omega +{\overset{―}{Y}}_{3x}\right)N_{3C}}{\omega +\left({\overset{―}{Y}}_{3x}\text{/}\hat{u}\right)}\; \end{array}$$ (12)

and then insert this equation for $N_{3E}$ into Eqn 11 to obtain


$$\begin{array}{c} \sigma_{F}\left(N_{3C}\right)=100\sqrt{T+\frac{1}{N_{3C}{\overset{―}{Y}}_{3x}}+\frac{\omega (\hat{u}+{\overset{―}{Y}}_{3x})}{{\overset{―}{Y}}_{3x}\left(e_{F}-\left[\omega +{\overset{―}{Y}}_{3x}\right]N_{3C}\right)}}. \end{array}$$ (13)

For a fixed amount of effort, we can now find the number of calibration fields of view that minimises this error ($N_{3C}^{*}$). This is a two-dimensional optimisation problem, and is calculated by taking the derivative of $\sigma_{F}$ with respect to $N_{3C}$ (Eqn 13) and setting it to zero:


$$\begin{array}{c} \frac{\partial }{\partial N_{3C}}\sigma_{F}\left(N_{3C}\right)=0\to N_{3C}^{*}(e_{F})=\frac{e_{F}}{\omega +{\overset{―}{Y}}_{3x}+\sqrt{\left(\omega +{\overset{―}{Y}}_{3x}\mathrm{\backslash rightleft}(\omega \hat{u}+{\overset{―}{Y}}_{3x}\right)}}. \end{array}$$ (14)

Substituting $N_{3C}^{*}\left(e_{F}\right)$ into Eqn 12, we can then find the corresponding optimal number of extrapolation-count fields of view ($N_{3E}^{*}$)


$$\begin{array}{c} N_{3E}^{*}(e_{F})=\frac{e_{F}\hat{u}}{\omega \hat{u}+{\overset{―}{Y}}_{3x}+\sqrt{\left(\omega +{\overset{―}{Y}}_{3x}\right)\left(\omega \hat{u}+{\overset{―}{Y}}_{3x}\right)}}. \end{array}$$ (15)

By dividing Eqn 15 by Eqn 14, we can provide the ratio of the optimal numbers of extrapolation-count ($N_{3E}^{*}$) to calibration-count ($N_{3C}^{*}$) fields of view. The sampling effort ($e_{F}$) cancels out, leaving us with the optimal field-of-view count ratio ($\delta^{*}$) in the following simple formula:


$$\begin{array}{c} \delta^{*}=\frac{N_{3E}^{*}(e_{F})}{N_{3C}^{*}(e_{F})}=\hat{u}\sqrt{\frac{\omega +{\overset{―}{Y}}_{3x}}{\omega \hat{u}+{\overset{―}{Y}}_{3x}}}. \end{array}$$ (16)

This is perhaps one of the most useful quantities for the practitioner of the FOVS method. It tells us that the primary variable in determining the most efficient number of field-of-view counts for a given assemblage is the ratio of target to marker specimens ($\hat{u}$). When $\hat{u}=1$, we have $\delta^{*}=1$, and so we should choose an equal number of calibration- and extrapolation-count fields of view. However, in the more typical scenario where there are greater numbers of targets than markers ($\hat{u}>1$), then the proportion of extrapolation-count fields of view should increase as the square root of $\hat{u}$.

The optimal field-of-view ratio ($\delta^{*}$) depends to a lesser degree on the density of target specimens across the study area (${\overset{―}{Y}}_{3x}$) and an observer’s counting habits (reflected by ω). Crucially, all three of these variables can be estimated during the calibration counts. To maximise the utility of the FOVS method for routine concentration estimates, we recommend utilising the relatively simple metric in Eqn 16 during the calibration counts for determining the optimal ratio of extrapolation- to calibration-count fields of view. The optimal field-of-view count ratio can be easily calculated by inserting the relevant variables directly into the user-friendly interface we have provided (link here: https://github.com/Palaeomays/FOVS_vs_linear_methods.git; additional information in S1 Text). Note that the optimal ratio of extrapolation- to calibration-count fields of view will not tell a practitioner how many of each should be counted for a given precision; this is discussed below (see ‘achieving a targeted precision’).

Lastly, if we wish to validly compare the efficiency of the two methods, we can now utilize the optimal field-of-view counts to characterise the FOVS method’s relationship between error vs effort. Substituting the optimal values $N_{3E}^{*}(e_{F})$ and $N_{3C}^{*}(e_{F})$ into Eqn 11 gives the FOVS method precision as a function of the effort


$$\begin{array}{c} \sigma_{F}\left(e_{F}\right)=100\sqrt{T+\frac{1}{e_{F}{\overset{―}{Y}}_{3x}}\left(\left[1+\hat{u}\right]\omega +{\overset{―}{Y}}_{3x}+\sqrt{\left[\omega +{\overset{―}{Y}}_{3x}\right]\left[\omega \hat{u}+{\overset{―}{Y}}_{3x}\right]}\right)\;}, \end{array}$$ (17)

which is analogous to the linear method error vs effort function in Eqn 10. In the supplementary material (see ‘analysis of precision as a function of effort and the ratio of common and rare grains’), we used S1 and S5 Eqns to show that the FOVS and linear methods both have the same “one on error-squared” decrease in error for increasing effort. So, we need to analyse the methods more closely to determine which is superior; these analyses are provided in the following section.

### Choosing the superior count method

The quality of a data collection method is measured by both its accuracy and its efficiency, the latter of which is the result of two competing variables: precision (σ) and sampling effort (e). Having optimised the ratio of extrapolation- to calibration-count fields of view for the FOVS method, we have now established standardised metrics of effort and error (=the inverse of precision) for both methods (Eqns 10 and 17). So, we can now directly compare the errors of the linear and FOVS methods for the same amount of effort (e). This will give the reader a formula for determining which method is the most efficient choice for their own studies.

By taking the ratio of the errors (for the FOVS method error [$\sigma_{F}$]; note, the optimal field-of-view counts [$N_{3C}^{*}$ and $N_{3E}^{*}$] are used), we obtain


$$\begin{array}{c} \frac{\sigma_{L}}{\sigma_{F}}=\sqrt{\frac{\omega \left(\hat{u}+1\right)+\left(2+Te+\hat{u}\right){\overset{―}{Y}}_{3x}+\left({\overset{―}{Y}}_{3x}\text{/}\hat{u}\right)}{\omega \left(\hat{u}+1\right)+(2+Te){\overset{―}{Y}}_{3x}+2\sqrt{\left({\overset{―}{Y}}_{3x}+\omega \right)\left({\overset{―}{Y}}_{3x}+\hat{u}\omega \right)}}}. \end{array}$$ (18)

If the value of this ratio is larger than one, then the FOVS method is expected to provide a smaller error for a given quantity of effort, while a value smaller than one would indicate that the linear method is superior. The point at which the ratio equals one is given by the solution to the following equation:


$$\begin{array}{c} {\left({\hat{u}}^{2}-1\right)}^{2}{{\overset{―}{Y}}_{3x}}^{2}-4\omega {\hat{u}}^{2}\left(1+\hat{u}\right){\overset{―}{Y}}_{3x}-4\omega^{2}{\hat{u}}^{3}=0. \end{array}$$ (19)

While this equation defines the dividing line between the choice of the two methods, we need not solve it. Instead, we can provide a simple formula for determining the appropriate method to use. The only parameters required for this determination are the assemblage-specific target-to-marker ratio estimate ($\hat{u}$) and the researcher-specific time parameter ω (the latter of which encompasses the time taken for field-of-view transitions and individual specimen counts). Given these data, we can then conduct a ‘method determination test’. The parameter below provides the minimum density of target specimens per field of view for which the FOVS method is the superior choice (${\overset{―}{Y}}_{3x}^{*}$):


$$\begin{array}{c} {\overset{―}{Y}}_{3x}^{*}=2\omega \frac{{\hat{u}}^{2}+\sqrt{{\hat{u}}^{3}\left(1+\hat{u}\left[\hat{u}-1\right]\right)}}{\left(\hat{u}+1\right){\left(\hat{u}-1\right)}^{2}}. \end{array}$$ (20)

So, if the mean number of target (or common) specimens per field of view for a given assemblage is larger than this number (i.e., ${\overset{―}{Y}}_{3x}>{\overset{―}{Y}}_{3x}^{*}$), then the FOVS method should be used; if it is smaller (i.e., ${\overset{―}{Y}}_{3x}<{\overset{―}{Y}}_{3x}^{*}$), then the linear method should be the preferred choice. Put another way, when specimen densities are low, a larger number of fields of view are needed to collect an accurate and precise data set, the time-cost of which disproportionately penalises the FOVS method. (Note: the subscript “x” for ${\overset{―}{Y}}_{3x}^{*}$ and ${\overset{―}{Y}}_{3x}$ denotes that the target specimens are the foci of the calibration counts; however, if the markers are the calibration count subjects, use the alternative equations in S1 Text).

### Achieving a targeted precision

Once we have settled on our method, we might like to obtain an estimate of the amount of effort required to achieve a desired maximum level of error. This desired error is denoted $\overset{―}{\sigma }$ (expressed in %).

For the linear method, we simply replace $\sigma_{L}$ with $\overset{―}{\sigma }$ in Eqn 10 and then rearrange it to find


$$\begin{array}{c} e_{L}\left(\overset{―}{\sigma }\right)=\frac{\omega \left(1+\hat{u}\right)+{\overset{―}{Y}}_{3x}\left(2+\hat{u}\right)+{\overset{―}{Y}}_{3x}\text{/}\hat{u}}{{\overset{―}{Y}}_{3x}\left({\left[\overset{―}{\sigma }\text{/}100\right]}^{2}-T\right)}. \end{array}$$ (21)

If, however, we have settled on the FOVS method for a given assemblage, then we would likely want to know how many calibration- and extrapolation-count fields of view would be required to achieve a targeted level of error. First, we start with Eqn 11 and then rewrite it using Eqn 16 to replace $N_{3E}^{*}$ as follows:


$$\begin{array}{c} \sigma_{F}\left(N_{3C}^{*},N_{3E}^{*}\right)=100\sqrt{T+\frac{1}{N_{3C}^{*}{\overset{―}{Y}}_{3x}}+\frac{1}{N_{3C}^{*}{\overset{―}{Y}}_{3x}}\sqrt{\frac{\left(\omega \hat{u}+{\overset{―}{Y}}_{3x}\right)}{\left(\omega +{\overset{―}{Y}}_{3x}\right)}}}. \end{array}$$ (22)

Using this equation, we solve for $N_{3C}^{*}$ to determine the optimal number of calibration-count fields of view for our desired error ($\overset{―}{\sigma }$):


$$\begin{array}{c} N_{3C}^{*}\left(\overset{―}{\sigma }\right)=\frac{1}{{\left(\overset{―}{\sigma }\text{/}100\right)}^{2}-T}\left(\frac{\sqrt{\left[{\overset{―}{Y}}_{3x}+\omega \right]}+\sqrt{\left[{\overset{―}{Y}}_{3x}+\hat{u}\omega \right]}}{{\overset{―}{Y}}_{3x}\sqrt{\left[{\overset{―}{Y}}_{3x}+\omega \right]}}\right). \end{array}$$ (23)

We can then use Eqn 16 again to obtain an expression for $N_{3E}^{*}$ for the optimal number of extrapolation-count fields of view for a given error ($\overset{―}{\sigma }$):


$$\begin{array}{c} N_{3E}^{*}\left(\overset{―}{\sigma }\right)=\frac{\hat{u}}{{\left(\overset{―}{\sigma }\text{/}100\right)}^{2}-T}\left(\frac{\sqrt{\left[{\overset{―}{Y}}_{3x}+\omega \right]}+\sqrt{\left[{\overset{―}{Y}}_{3x}+\hat{u}\omega \right]}}{{\overset{―}{Y}}_{3x}\sqrt{\left[{\overset{―}{Y}}_{3x}+\hat{u}\omega \right]}}\right). \end{array}$$ (24)

**Fig 4:**
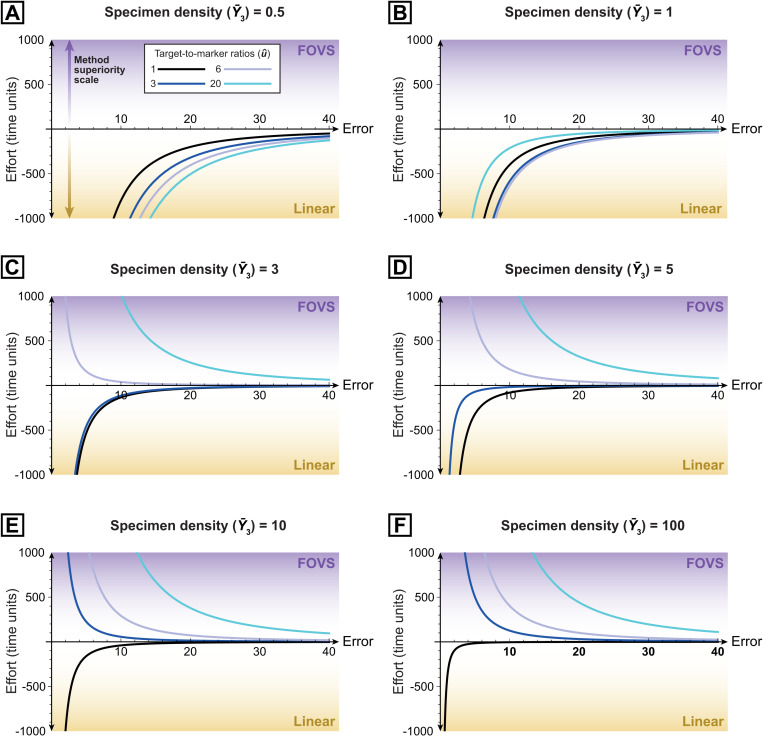
Residual efficiencies of the linear vs FOVS data collection methods, and the influences of specimen density (${\overset{―}{Y}}_{3}$) and target-to-marker ratio ($\hat{u}$). These plots illustrate the amount of net effort required for the linear method compared to the FOVS method (with optimal field-of-view counts; Eqn 26), where FOVS method data uses the optimal number of calibration- and extrapolation-count fields of view (Eqns 23 and 24). Hence, negative values indicate cases where the linear method has superior efficiency (with lower values indicating cases of even greater relative efficiencies for the linear method); the FOVS method is more efficient in all other cases. In all cases, error contribution from the marker doses (T) is zero, and the ratio of field-of-view transition to specimen count time (ω) = 2. (A) ${\overset{―}{Y}}_{3}=0.5$. (B) ${\overset{―}{Y}}_{3}=1$. (C) ${\overset{―}{Y}}_{3}=3$. (D) ${\overset{―}{Y}}_{3}=5$. (E) ${\overset{―}{Y}}_{3}=10$. (F) ${\overset{―}{Y}}_{3}=100$.

The above equations (Eqns 23 and 24) tell us the number of fields of view needed for a specific level of error. To arrive at a prediction for the amount of effort required to achieve that error, we substitute Eqn 23 into Eqn 13 and rearrange to find:


$$\begin{array}{c} e_{F}\left(\overset{―}{\sigma }\right)=\frac{2{\overset{―}{Y}}_{3x}+\omega \left(1+\hat{u}\right)+2\sqrt{\left({\overset{―}{Y}}_{3x}+\omega \right)\left({\overset{―}{Y}}_{3x}+\hat{u}\omega \right)}}{{\overset{―}{Y}}_{3x}\left({\left[\overset{―}{\sigma }\text{/}100\right]}^{2}-T\right)}, \end{array}$$ (25)

which can be compared to the analogous expression for the linear method (Eqn 21). This comparison is done simply by calculating the difference between the two methods:


$$\begin{array}{c} e_{L}\left(\overset{―}{\sigma }\right)-e_{F}\left(\overset{―}{\sigma }\right); \end{array}$$ (26)

this gives us another way to compare the efficiencies of the two methods (in addition to the function expressed in Eqn 20). Negative values for Eqn 26 indicate that the linear method would be more efficient, while positive values indicate that the FOVS method should be used (Fig 4).

Of the variables included in Eqn 25, we can see that both target-to-marker ratio ($\hat{u}$) and target specimen density (${\overset{―}{Y}}_{3}$) have major roles in determining the most appropriate method. ${\overset{―}{Y}}_{3}$ has a disproportionately large impact on the efficiency of the FOVS method; for assemblages with extremely low target densities (hence, very small ${\overset{―}{Y}}_{3x}$), the linear method is always the best choice (Fig 4C), otherwise too much time is spent on field-of-view transitions. In the case of microfossils, however, in our experience, assemblages with such low densities are rare, and indicative of poor preparation and/or poor fossil recovery. For assemblages with more reasonable (moderate to high) target specimen densities, then the linear method is only the best choice when there is roughly the same proportion of targets and markers (i.e., $\hat{u}\approx 1$; Fig 4A, B; S1 Fig), and even then, only marginally. In all other cases, the FOVS method is superior.

As noted above (see ‘choosing the superior count method’), the most efficient method can be determined with very few input parameters (Eqn 20). Eqn 26 also provides this determination, while quantifying how much more efficient that method is over the other (for a given precision; Fig 4). Should the FOVS method be the superior choice, Eqns 23 and 24 provide the required number of fields of view for the calibration counts and extrapolation counts, respectively, for a desired precision level. For routine data collection, these equations will inform users about the feasibility of achieving satisfactory precision for each assemblage.

## Software interface for calculating parameters of the linear and FOVS methods

We devised an alternative, interactive way for users to calculate the quantitative parameters described in this paper. The ‘absolute abundance calculator’ is a user-friendly interface that provides all the practical outputs for these methods, including concentration, uncertainty and effort. Moreover, the calculator assists in determining the most efficient method (either linear or FOVS) for a given sample, and the optimal numbers of fields of view (FOVS method).

The calculator has been designed using Microsoft Excel’s Visual Basic for Applications (VBA). It uses macros—predefined sets of instructions that can be executed automatically—that help users with certain tasks, such as automatically determining variables (e.g., ω, $s_{3}$, or the optimal numbers of calibration- and extrapolation-count fields of view [$N_{3C}^{*}$ and $N_{3E}^{*}$, respectively]), a timer, a counting assistant that allows faster counting (i.e., more efficient data collection) analogous to physical methods (e.g., tally counters), and a way to export all variables and counts to spreadsheets.

This ‘absolute abundance calculator’ is open-source and hosted in a GitHub repository, where its VBA code can be read, and the application downloaded. Software updates will be made available there, and users are free to use GitHub functionalities as a collaborative platform to raise any issues or suggest code changes.

The application is contained inside a single macro-enabled Microsoft Excel worksheet (file format extension: xlsm). Users are required to first enable macros to run this tool. Additional instructions, a walkthrough and worked example are present in S3 File and the README.md file present in the GitHub repository. Readers can access the ‘absolute abundance calculator’ here: https://github.com/Palaeomays/FOVS_vs_linear_methods.git.

## Case studies

The precisions and efficiencies of the two count methods were analysed with two case studies, each of which followed an independent paradigm: 1, computer simulation; and 2, ‘real-world’ microfossil assemblage data. Three outcome variables were examined and compared between the two counting methods: 1, absolute abundance (concentration; Eqns 1 or 4); 2, error (= the degree of uncertainty to which the true concentrations in an assemblage can be inferred from a count; Eqns 2 or 5); and 3, data collection effort (Eqns 6 or 7).

### Case study 1—Computer simulations: Methods

The purpose of this simulation was to produce a series of idealised data sets to compare the precisions of the traditional ‘linear count method’ against the new ‘FOVS method’ for an equivalent amount of effort. For this, we used a Monte Carlo simulation of ‘virtual study areas’ containing targets and markers. Monte Carlo simulations are a method of generating and testing hypotheses for complex systems using a large number of random instances of the scenario being studied. The simulation parameters were designed to mimic the (approximately) random distributions of specimens on a virtual study area; e.g., microfossils on microscope slides. Microscope slides are a small, well-controlled sample of approximately random particles, which are time- and labour-intensive to analyse in the laboratory, and so they are ideal candidates for a Monte Carlo approach. However, the simulated and microfossil case studies have subtly different assumptions: the former assumes random (Poisson) distributions, while the latter merely assumes equivalent distributions of targets and markers, but these are not necessarily random (see discussion in the ‘field-of-view subsampling method (‘FOVS method’): limitations’ section).

**Fig 5:**
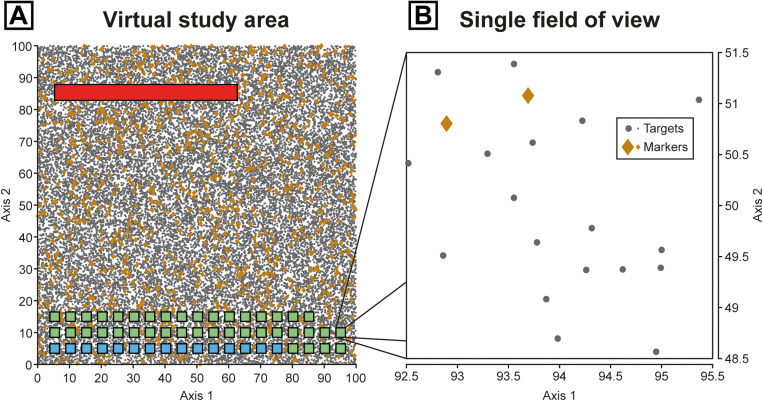
Visualisations of a virtual study area to test the precision of the linear vs FOVS methods for a given amount of collection effort. It includes the random distribution of 30,000 target specimens (grey dots) and 1,000 markers (brown diamonds); hence, $\overset{―}{\overset{―}{u}}$ = 30. (A) Full virtual study area, red rectangle is the “window” used for the linear method data, squares are examples of the individual fields of view used for the FOVS method: blue are for the calibration-count fields of view, green are the extrapolation-count fields of view. (B) A representative field of view for the calibration or extrapolation counts used in the FOVS method.

One benefit of these Monte Carlo simulations is that for each virtual study area, we can specify exact specimen population sizes. For each sequence of iterations of the simulation, the total number of target specimens ($\overset{―}{\overset{―}{x}}$) was fixed (= 30,000) and these were distributed uniformly at random on a square virtual study area (Fig 5A). Between each sequence of iterations, however, the total number marker specimens ($\overset{―}{\overset{―}{n}}$; also distributed uniformly at random on the same study area) were varied to test for the effects of target-to-marker ratio ($\hat{u}$). To validly compare the two methods, the count parameters (i.e., the counted targets [x], markers [n] and fields of view [$N_{3C}$, $N_{3E}$]) were modified for each sequence of iterations to ensure near-constant sampling efforts between sequence and between methods.

Monte Carlo simulations allow us to calculate quantities not possible in most real-world study areas. For example, both the simulation and empirical approaches (see ‘case study 2—organic microfossils: methods’) enable the comparison of method precision, reflected by their standard errors. However, the simulations can also measure the accuracy of each method’s error estimates, indicated by variations of the error estimates from the known true values (see S4–S14 Tables, S1 Fig).

Since we are comparing the linear method to the FOVS method on the same data sets, the error contribution from the addition of marker doses ($T={s_{1P}}^{2}\text{/}N_{1}$) was the same for both methods. So, for the simulations to provide the clearest comparisons of the two methods, we have specified the number of markers exactly. In other words, there was no error contribution from the exotic markers. This gives us a precise value of $N_{1}\times {\overset{―}{Y}}_{1}\;$ in Eqns 1 and 4, and consequently results in $s_{1P}=0$ (see Eqns 2 and 5). Additionally, we set the total sample dimension size ($\overset{―}{V}$) as 1 arbitrary size unit; this parameter is analogous to the total mass or volume of a sediment from which microfossils might be counted. Lastly, the field-of-view transition effort factor (*ω*) was constant for all iterations (= 2).

We then applied the following procedures to each virtual study area.

#### Linear method.

Starting at a fixed point on the virtual study area, a contiguous region of pre-determined height and variable length was marked out (Fig 5). The length was modified for each virtual study area to encompass a specified number of target specimens (x). This provided our random “window” within which the number of markers (n) were also counted. These data were then used to produce the following simulated parameters of the linear method: target specimen concentration ($c_{L}$; Eqn 1), standard error ($\sigma_{L}$; Eqn 2) and sampling effort ($e_{L}$; Eqn 6).

#### FOVS method.

Firstly, an initial FOVS method sampling effort ($e_{F}$) was chosen that was as close as possible to the linear method sampling effort ($e_{L}$) of the same virtual study area. This level of effort was used to estimate the optimal numbers of calibration fields of view ($N_{3C}^{*}$) and extrapolation-count fields of view ($N_{3E}^{*}$) for the FOVS method (see ‘FOVS method optimisation’ above for details). Then the total number of common specimens was counted from this optimal number of calibration-count fields of view, from which we calculated the sample mean (${\overset{―}{Y}}_{3}$) and proportional sample standard deviation ($s_{3P}$) of the common specimens. (Note: in all simulated cases, the number of targets was equal to or greater than markers [x≥n]; so, the targets [x] were the foci of the calibration counts, and ${\overset{―}{Y}}_{3}={\overset{―}{Y}}_{3x}$.) Next, the number of rare specimens (= markers [n] in all simulated cases) were counted from the corresponding optimal number of extrapolation-count fields of view ($N_{3E}^{*}$). With these data, we then calculated the following parameters for the FOVS method: target specimen concentration ($c_{Fx}$; Eqn 4) and standard error ($\sigma_{\text{F}x}$; Eqn 5) and the sampling effort ($e_{F}$; Eqn 7).

Note that we experimented with the parameters for the linear method, so that the level of effort required for the FOVS method kept the total number of fields of view to a suitably small number, specifically $1\leq N_{3C}+N_{3E}\leq 361$. (Our simulated virtual study areas can have a maximum of 1089 non-overlapping fields of view.) By keeping the maximum sampled area much smaller than the total study area, we can avoid significant “finite population effects”, whereby too many samples from a finite area will produce distorted statistical estimates. In the extreme case, if the total sample count contains the entire population, then the sample standard deviation will be zero. To ensure we make precise comparisons between the estimates from the two methods (linear and FOVS) with the computer simulations, we included a “finite population correction” (FPC; see [64], p. 83; details in S1 File).

#### Simulated data sets.

For each set of parameters, we ran the simulation 1,000,000 times, producing data sets of the concentration, total error and effort for each iteration of both methods (see S4–S14 Tables). We then computed the mean values of: 1, the estimated concentrations (c; Eqn 1); 2, the total errors ($\sigma_{\text{L}}$ and $\sigma_{\text{F}}$; Eqns 2 and 5), recalling that we have no error contribution from the marker doses; 3, the standard deviation from the known exact concentrations ($\sigma_{exact,M}$; S17 Eqn); and 4, the required data collection effort ($e_{L}$ and $e_{F}$; Eqns 6 and 7). For maximum precision of the simulations, additional statistical corrections and effort standardisation across the different methods were included (see S1 File).

The simulations were generated in Matlab v.R2020a. The code to generate these simulations (S2 File), and instructions on how to implement and modify the code, can be downloaded here: https://github.com/Palaeomays/FOVS_vs_linear_methods.git. For each instance of the simulation, the user chooses:

- the number of targets and markers on the slide ($\overset{―}{\overset{―}{x}}$ and $\overset{―}{\overset{―}{n}}$, respectively);
- the error contribution from the marker doses ($s_{1}$);
- the number of effort units (e);
- the field of view transition factor (ω); and
- the number of iterations of the simulation.

### Case study 1—Computer simulations: Results

**Fig 6:**
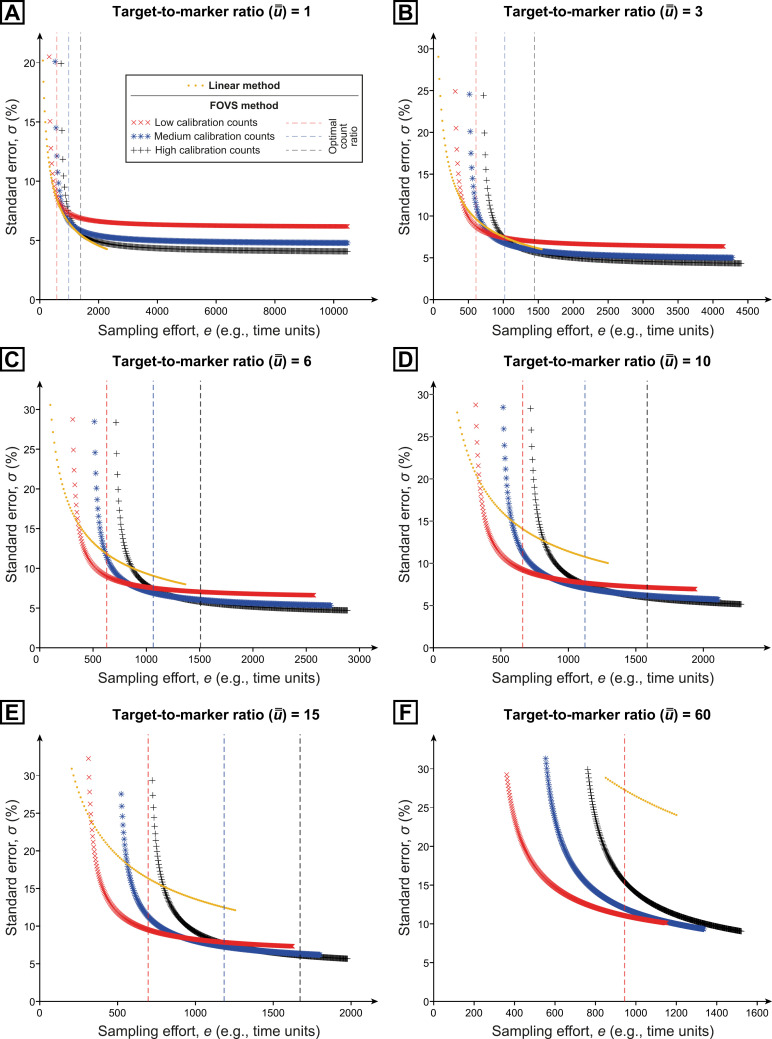
Simulated data sets showing the relationships between precision and effort using the linear method (orange) vs the field-of-view subsampling (FOVS) method. To test the effects of field-of-view count ratios, the three FOVS method plots represent three different count conditions; each condition had a fixed number of calibration-count fields of view ($N_{3C}$), and effort was incrementally increased by sequentially adding extrapolation-count fields of view ($N_{3E}$); red: $N_{3C}=10$ and $1\leq N_{3E}\leq 351$; blue: $N_{3C}=14$ and $1\leq N_{3E}\leq 344$; black: $N_{3C}=21$ and $1\leq N_{3E}\leq 337$. The dashed lines indicate the optimum ratio ($\delta^{*}$ in Eqn 16) of extrapolation- to calibration-count fields of view for each of the three FOVS count conditions. (Note that for $\overset{―}{\overset{―}{u}}=60$, only the red dashed line appears, as there were not enough extrapolation-count fields of view in this simulation to reach the optimal ratio for the other two [blue and black] count conditions). For the linear method, effort was gradually increased by lengthening the “window” until a predetermined number of targets was counted. Error contributions from the markers have been set to zero (T=0); hence, absolute errors will be underestimates of those expected in a natural sample, but the relative errors between methods will be accurate. Each simulated scenario underwent 100,000 iterations. Six different scenarios are provided, which reflect different target-to-marker ratios ($\overset{―}{\overset{―}{u}}$). (A) $\overset{―}{\overset{―}{u}}=1$. (B) $\overset{―}{\overset{―}{u}}=3$. (C) $\overset{―}{\overset{―}{u}}=6$. (D) $\overset{―}{\overset{―}{u}}=10$. (E) $\overset{―}{\overset{―}{u}}=15$. and (F) $\overset{―}{\overset{―}{u}}=60$.

The simulated error vs effort relationships (summarised in Fig 6) demonstrate the following:

1) For both methods, a target-to-marker ratio ($\hat{u}$) of 1 produces the lowest error.
2) With near-equivalent numbers of markers and targets ($\hat{u}\approx 1$), both FOVs and linear methods require similar efforts to achieve errors of < 8% (excluding error associated with the introduction of exotic markers; e.g., quantities of *Lycopodium* spores per tablet).
3) At $\hat{u}$ values close to 1, the linear method is slightly more efficient. However, even with a moderate difference between an assemblage’s target and marker abundances—especially $\hat{u}\geq 3$—the amount of effort required for the linear method is consistently higher than the FOVS method for the same degree of precision (regardless of the FOVS method calibration count size).
4) In addition to the principal role that $\hat{u}$ plays in determining method choice, there is a non-trivial dependence on the mean density of targets in a field of view (${\overset{―}{Y}}_{3x}$). At moderate to high values of ${\overset{―}{Y}}_{3x}$, the FOVS method is more efficient; at extremely low ${\overset{―}{Y}}_{3x}$, the linear method is superior (all other variables being equal).
5) The simulated case study enabled exact concentrations and errors to be calculated, against which the estimated total errors of both linear and FOVS methods could be compared (S1 File; S4–S14 Tables). From these comparisons, it was clearly demonstrated that the error estimates for the FOVS method are extremely accurate, regardless of $\hat{u}$, and consistently superior to the linear method; the linear method precision estimates, in contrast, are particularly unreliable at high $\hat{u}$ values (S1B Fig).
6) The minor discrepancies in total error from the three different FOVS scenarios are largely due to the small standard deviations derived from the calibration counts ($s_{3}$). This reflects the low spatial heterogeneity in the randomly distributed simulated assemblage. Under such scenarios, it is far more efficient to collect a smaller number of calibration counts. However, if the specimen density of the sample is particularly heterogeneous, then the error of these calibration counts will be proportionally high, which will then require a higher effort to achieve a desired precision. (A systematic investigation of spatial heterogeneity effects is beyond the scope of the present study.)

### Case study 2—Organic microfossils: Methods

#### Processing, imaging and sample details.

**Fig 7:**
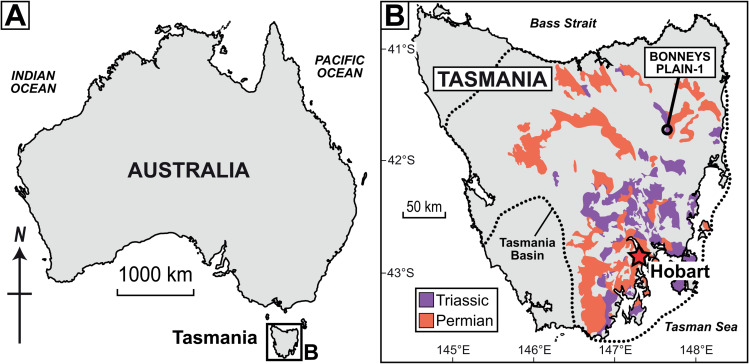
Geographic and geologic contexts for the Permian–Triassic organic microfossil assemblages. (A) Map of Australia. (B) Geological map of the Tasmania Basin with location of target well core succession (Bonneys Plain-1) and approximate distributions of Permian-Triassic sedimentary strata ([70]; 1:500,000 Digital Geological Data reproduced from Mineral Resources Tasmania (http://www.mrt.tas.gov.au/) under a Creative Commons Attribution 3.0 Australia license, © State of Tasmania).

All empirical data analysed herein derive from one drill core within the Tasmania Basin, southeastern Australia: Bonneys Plain-1 (41° 46’ 27.69“S, 147° 36’ 13.35”E; Fig 7). The target strata were part of the upper Parmeener Supergroup and, although these strata lack precise age control, they correlate to the upper Permian (Lopingian) to Lower Triassic Series [71]. For organic microfossil processing and comparisons of linear and FOVS count methods, 18 samples were chosen at random stratigraphic heights throughout the drill core (see S3 Table for details). The assemblages consisted of a range of shallow marine to coastal plain palaeoenvironments ([71]; C.R. Fielding, pers. comm.). Inorganic mineral content was removed by digestion with hydrochloric acid followed by hydrofluoric acid. Prior to acidification (following [51]), a number of tablets of *Lycopodium clavatum* spores were added; these spores served as the exotic markers in this case study. Tablets were produced by the Department of Geology, University of Lund, Sweden. Details of the tablets (including means and uncertainty estimates of *Lycopodium* spores per tablet and batch numbers), the specific quantities of *Lycopodium* tablets ($N_{1}$) added to each sample and spore quantity estimates of each tablet (${\overset{―}{Y}}_{1}$, $s_{1}$, $s_{m}$) are provided in S3 Table. No sieving, heavy liquid separation or oxidation was performed for these ‘kerogen’ residues. The resultant residues were mounted on glass slides, and glass coverslips were sealed with epoxy. A summary of these various aspects of palynological processing techniques was provided by Riding [72]. These residues of ‘sedimentary organic matter’ (*sensu* [73]) are the targets of palynofacies analysis (*sensu* [74]) and are reflective of the undissolved particulate organic carbon (*sensu* [75,76]) of a sediment sample’s ‘total organic carbon’ (or TOC).

By targeting mudrock facies of continental environments (e.g., lacustrine, fluvial, coastal), these microfossil assemblages were likely the result of low-energy, suspension deposition proximal to their sources (e.g., [77–81]). This is reflected by the high proportions of plant-derived phytoclasts in most assemblages. Moreover, estimating microfossil concentrations from sedimentary organic matter assemblages should avoid sieving, oxidation and heavy liquid separation steps, each of which has strong potential for introducing unintentional biasing effects ([49]; see reviews [72,82]).

These samples were processed by Global Geolab Limited in Medicine Hat, Canada. Transmitted light microscopy and photomicrography of organic microfossils was conducted with a Zeiss Axioskop 2 transmitted light microscope equipped with a Canon EOS 700D camera. Fluorescence photomicrographs were performed with a Leica K3C camera on a Leica DM2500 LED microscope. Residues and slides have been given the prefix ‘S’ and are housed at the Department of Palaeobiology, Naturhistoriska riksmuseet (NRM), Stockholm, Sweden. The rock core samples from which the microfossil assemblages were derived were collected from the Mornington Core Library, Mineral Resources Tasmania (MRT), Australia. All necessary permits were obtained for the described study, which complied with all relevant regulations; specifically, permission for rock core sampling was provided by MRT. Additional specific methodological details for the different concentration estimates discussed are provided below where relevant.

#### Count methods.

In these assemblages, the principal goal was to estimate the concentration of terrestrial organic microfossils ($c_{t}$). To this end, terrestrial organic microfossil grains were the target specimen type (x) and the subjects of the calibration counts; spores of *Lycopodium clavatum* were the exotic marker grains (n). The terrestrial organic microfossil populations consisted of (in order of approximate decreasing abundance): wood (=’phytoclasts’, including charcoalified wood), leaves, plant spores, pollen and fungal remains. Fossil resins and animal-derived clasts were negligible. For both count methods, only grains ≥5 μm in diameter were counted; specimens smaller than this could not be consistently identified.

**Fig 8:**
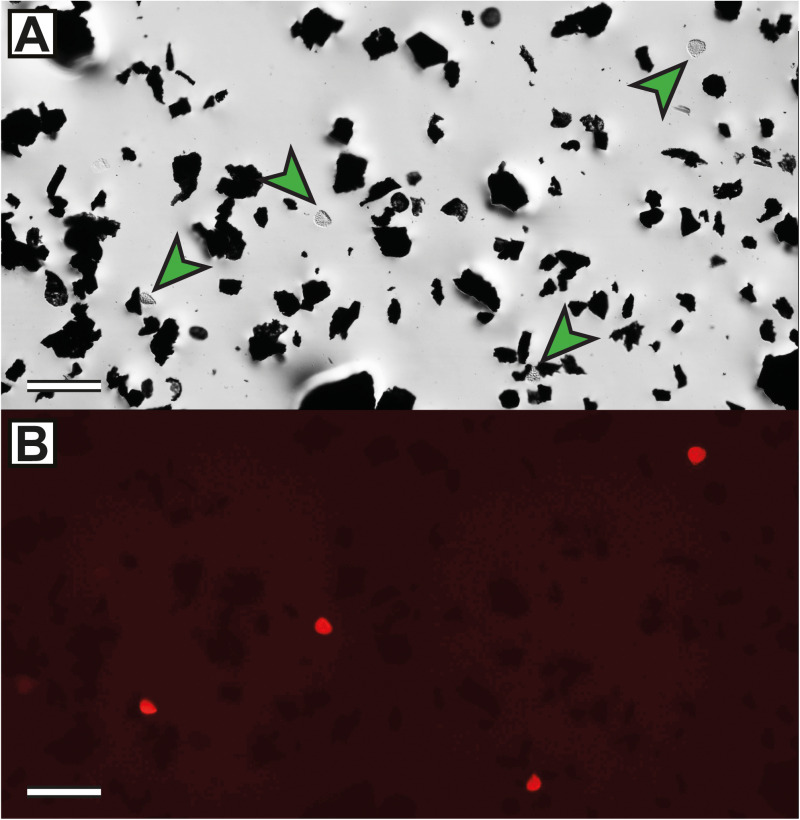
A typical rectangular field-of-view photomicrograph of an organic microfossil (‘palynological’) assemblage spiked with *Lycopodium* spore markers. Specimen S090248, well core MPT-3, Tasmania Basin; scale bars = 100 μm. (A) Greyscale optical light microscopy (with differential interference contrast). (B) Fluorescence microscopy (excitation wavelength: 555 nm); note the strong autofluorescence of the *Lycopodium* markers (green arrows).

The assemblages in this case study had the following criteria that made them particularly suitable to the FOVS method of data collection: 1, the *Lycopodium* spores were optically distinct from the other microfossils (e.g., colour, texture, transparency, fluorescence response), even from fossil spores or pollen (Figs 2B, 8); and 2, generally, one of these grain populations was relatively rare (mean target-to-marker ratio for Bonneys Plain-1 was high: $\hat{u}>10$). All fields of view were examined with a 63 × magnification objective. The field-of-view transition effort factor (ω) was measured as approximately twice that of counting a single specimen on one field-of-view (i.e., ω=2). All assemblages included a sampling effort (see below) of > 500. To minimise observer expectation biases, all counts followed the “blind protocol” outlined by Mays & McLoughlin ([30], p. 297); specifically: 1, all slide labels were masked; 2, slide order was randomized; and 3, sample counts were then conducted.

### Case study 2—Organic microfossils: Results

When the linear and FOVS methods were applied to the Permian–Triassic terrestrial organic microfossil assemblages of the Tasmania Basin, the concentration estimates ($c_{t}$) produced by the two methods were very similar, with the FOVS method tending towards slightly higher values (Table 1). A paired *t*-test was used to test whether the differences in mean $\;c_{t}$ between these two methods were statistically significant. The paired test was essential since the two populations consisted of random samplings using different count methods, but taken from the same populations (organic residues of the same sedimentary rock sample); the null hypothesis predicted no difference in $c_{t}$. The paired *t*-test revealed a non-significant difference between the mean $c_{t}$ values for these methods (*t* = 1.893; *p* = 0.08; *N* = 18). An illustration of this concordance: in all assemblages, the concentration estimates from the FOVS method counts fell within the 95% confidence intervals of the linear method counts (S3 Table).

**Table 1:** Comparison table of terrestrial organic microfossil concentrations ($c_{t}$), and their associated uncertainties (total standard errors) and sampling efforts.

Parameter	Linear method	Field-of-view subsampling (FOVS) method
Concentration of terrestrial organic microfossils ($c_{t}$, specimens/g), mean	328478^a^	344341^a^
Total standard error (σ, %), mean	± 29.1^b^	± 11.65^b^
Sampling effort (e), mean	990	1064
Calculated target-to-marker ratio ($\hat{u})$, post-count mean	89.8	94.3
Predicted effort ($e[\overset{―}{\sigma }]$, in time units) for a target of 10% error, post-count mean	10069	1445

Samples (*N* = 18) are from the Permian–Triassic assemblages of Bonneys Plain-1, Tasmania Basin, Australia (see S3 Table).

a No statistically significant difference (*p *> 0.05).

b Highly statistically significant difference (*p *< 0.0005).

The differences in precision between the two methods, however, were pronounced, as reflected by their total error values ($\sigma_{\text{L}}$ and $\sigma_{\text{F}}$). A Wilcoxon signed-rank test was employed to test whether the differences in median total error between the two methods were statistically significant. This non-parametric test was chosen because it applies to paired samples, but does not assume normally distributed data [83,84]. The null hypothesis predicts zero differences between the medians. The FOVS method resulted in a much lower total error; this difference was highly statistically significant (*W* = 171, *p* = 0.0002; *N* = 18). In principle, this high significance value is typical of: 1, a modest variation in precision (i.e., a modest ‘effect size’) for a large sample set; or 2, a major effect size from only a small sample set. Since there was only a small number of assemblages in the comparisons (*N* = 18), this indicates a major ‘effect size’; specifically, a major improvement in precision for the FOVS method. Crucially, this precision improvement was despite a similar mean sampling effort (Table 1). Given that the two methods were conducted on identical samples, the calculated difference in mean concentration estimates (c. 6%) was likely due in large part to the relatively large error estimates of the linear method. Lastly, we utilised Eqns 23 and 24 ($e_{L}[\overset{―}{\sigma }]$ and $e_{F}[\overset{―}{\sigma }]$, respectively) to predict the sampling efforts required by the two methods to achieve the same error rate. To achieve an error of 10%, the linear method would require approximately 7 times the sampling effort than the FOVS method (Table 1).

## Discussion

### Absolute abundance data collection techniques

The two methods employed herein yielded similar absolute abundances (or concentrations) in both simulated and empirical data sets. However, the quality of a new method is also demonstrated by its ability to produce the same or higher precision, for the same or lower sampling effort. Moreover, the superior method should provide a more accurate approximation of its precision. Despite the benefits of the ‘field-of-view subsampling method’ (FOVS method), including greatly decreased error rate with consistent sampling effort, there are important limitations to this technique that need to be balanced against the strengths and limitations of the linear method. The limitations of each method are expanded below and summarised in Table 2.

**Table 2:** Summary of applicability for the two absolute abundance count methods discussed herein.

Consideration	Linear method	Field-of-view subsampling (FOVS) method
Precision sensitivity to target-to-marker ratio ($\hat{u}$)	High	Low to moderate
Optimal range of target-to-marker ratio ($\hat{u}$) values^a^	c. 0.33–3	c. 0.05–20
Accuracy of error estimates	For $\hat{u}\approx 1$	For all tested values of $\hat{u}$
Concentrations of multiple target populations ($c_{1},c_{2},\ldots ,\;c_{k}$)?	No^b^	Yes
Simultaneous collection of relative abundance data?	Yes	No
Precision sensitivity to specimen heterogeneity^c^	Low	Moderate to high
Sampling effort sensitivity to specimen density (${\overset{―}{Y}}_{3}$)	Low	Moderate

^a^ While maximum count precision is at $\hat{u}=1$ [61], the optimal efficiency of each method is at $\hat{u}\approx 2$ (with target counts of ≥ 500; [31]).

^b^ It is very unlikely that near-optimal target-to-marker ratios of more than one target population will co-occur in a given assemblage.

^c^ This factor has not been rigorously explored in this study.

#### Linear method: Limitations.

The key limitation of the linear method lies in its high sensitivity to the ratio of target specimens to introduced markers; i.e., the ‘target-to-marker ratio’ ($\hat{u}$). Regardless of the count type, the highest precisions are achieved when the target-to-marker ratio is close to 1:1 [31,61]. We have demonstrated that, in these optimal conditions (and with reasonable values for other relevant parameters), the linear method shows a marginally superior efficiency (Figs 4 and 6). However, the linear method has a low tolerance for ratio values far beyond this [49,51,85]. This has important practical limitations:

A. ***Reprocessing of samples:*** For the microfossil assemblages discussed herein, the exotic markers are introduced during sample preparation; hence, before observation at the microscope [72]. However, it is impossible to accurately predict *a priori* how many markers to add without examining the prepared microfossil assemblages for approximate concentrations of the target specimens. Attempts to circumvent this apparent paradox typically involve undertaking two (or more) preparation phases. For instance, the preparator might: firstly, predict the optimal number of markers to introduce for a given sample (often informed by sedimentary indicators of microfossil concentrations, e.g., sediment grain-size or colour, inferred depositional conditions, total organic carbon analyses); secondly, conduct a preliminary batch of processing; thirdly, examine a subsample of the assemblage to gauge the approximate target-to-marker ratio and, if the ratio is suboptimal, conduct a subsequent phase of processing with a modified number of introduced markers. This may entail several processing rounds. Repeated processing can be costly in time, money and sediment (or sedimentary rock) sample, the latter of which may be irreplaceable; hence, this may be impractical for many potential applications. In principle, processing costs might be mitigated by adding the markers towards the end of the preparation procedure, as this would reduce the number of steps during reprocessing (e.g., acidification, oxidation; [49,50]). However, the late-stage introduction of exotic markers can lead to their overrepresentation, because the early stages of sample preparation demonstrably bias the assemblages [51]. Hence, marker grains should be introduced at the start of the preparation process, resulting in a consistent degree of bias for both markers and targets [51], but also leading to a longer and more costly preparation should the sample need to be processed multiple times.
B. ***High data collection effort:*** In cases with suboptimal target-to-marker ratios, the key limiting factor in concentration precision will be the low abundances of the rare specimens (typically, these will be the exotic markers). Thus, increasing the number of the rare specimens counted, even by a modest amount, can provide an enormous increase in precision. The ‘brute force’ way to bolster the count of these is simply to undertake a longer data collection phase. However, since precision is the result of multiple factors (Eqns 2 and 5), the relationship between target-to-marker ratios and effort is nonlinear. We have shown (S1 Eqn) that the increase in precision decreases with the square of the effort. Thus, assemblages with higher ratios require inordinate increases in specimen counts—therefore, data collection effort—to achieve the same low degree of uncertainty than assemblages with low ratios ([31,34]) (Fig 6). For example, if seeking a total error of 10% with a target-to-marker ratio of 3:1 ($\hat{u}=3$), a density (${\overset{―}{Y}}_{3x}$) of 10 targets per field of view, and assuming similar exotic marker errors (T) and transition-to-count ratios (ω) as those employed in this study, one would need a total effort of c. 627 effort units (calculated from Eqn 21). However, with a target-to-marker ratio of 20:1 ($\hat{u}=20$), but all other parameters being equal, an effort of c. 2682 is required to achieve the same degree of precision. Under such conditions, it is entirely reasonable to reprocess the samples. As stated by Maher ([31], p. 188): “it requires very little effort to add a few marker tablets to some sediment [samples]. It *does* require effort to increase the pollen counts.”
C. ***Unsuitable for multiple concentration targets:*** A limitation related to the linear method’s sensitivity to target-to-marker ratios is that an ‘optimal’ ratio assumes only one target population. However, there may be more than one target population (e.g., $x_{1},x_{2},\ldots ,\;x_{k}$) for which absolute estimates are being sought, each of which will have its unique concentration value (e.g., $c_{1},c_{2},\ldots ,c_{k}$). An assemblage with an ideal target-to-marker ratio for one target type (e.g., pollen, algae, terrestrial organic microfossils) is unlikely to be suitable for other potential target types in the same assemblage, given the narrow window of target-to-marker ratio suitability for the linear method (which, ideally, should be close to $\hat{u}=1$). Hence, the chances of achieving satisfactory concentration precisions for multiple targets using the linear method becomes vanishingly small (without reprocessing for each target population [see point 1 above] or extensive data collection effort [see point 2 above]).

For the reasons above, there will be many instances when either the targets or exotic markers are disproportionately rare. This will result in low precision, reflected by large total error values and confidence intervals. It is worth noting that even in extreme cases where the target-to-marker ratios are exceptionally high or low, the linear method can still inform a qualitative assessment even with large error values. A very high (or very low) target-to-marker ratio is indicative of a very high (or very low) absolute abundance of targets. In this case, the linear method may provide an approximate minimum (or maximum) value for these abundances (e.g., [86]). However, most studies would require more precise estimates. With the linear method, collecting sufficient count data to improve these precisions may require reprocessing, or an inordinate data collection time, both of which may be prohibitive for routine study. The FOVS method aims to improve precision and/or sampling effort for samples with suboptimal target-to-marker ratios.

#### Field-of-view subsampling (FOVS) method: Limitations.

The resilience of the FOVS method to the target-to-marker ratio circumvents a key limitation of the linear method. As a result, very large, extrapolated data sets can be obtained using the FOVS method, while bolstering the number of rare specimens. Despite the compounded error that this extrapolation entails, our simulated and empirical case studies (see the ‘case study 1—computer simulations: results’ and ‘case study 2—organic microfossils: results’ and sections, respectively) demonstrate generally improved precision for absolute abundance estimates and/or decreased data collection effort. Furthermore, the simulations show that the FOVS method yields a more accurate approximation of precision. However, some limitations should be considered before applying this technique:

1. ***Unsuitable for relative abundances:*** The FOVS method is designed for absolute abundance (e.g., concentration) estimates. By counting entire fields of view in a rapid sequence (during the extrapolation-count phase), it extrapolates large data sets by bypassing the collection effort of individual identifications. In contrast, a primary strength of the linear method is the identification of all target specimen types. A byproduct of the latter approach is a robust quantitative data set of the indigenous specimen populations. Hence, the linear method has the potential to provide concentrations for only a small number of targets (those with near-optimal target-to-marker ratios) while providing accurate, concurrent collection of compositional data (or relative abundance data) for the organic constituents of a sediment or sedimentary rock. Such relative abundance data form a cornerstone of pollen [87] and palynofacies [88] analyses. However, depending on the research question, the time-consuming determination of relative population abundances may not be necessary if absolute abundances are the primary goal.
2. ***Requires visual contrast:*** In visual observation applications, the FOVS method would work best when there is sufficient visual contrast between exotic markers and the indigenous specimens in the assemblage (Fig 2B). This contrast enables rapid and accurate identification of markers during the extrapolation counts. Without this, thorough observation of each field of view is required to produce accurate results, thus increasing data collection time (specifically, modifying ω). The most common exotic markers utilized in organic microfossil studies today, modern *Lycopodium* spores, have typically undergone acetolysis during preparation which darkens and discolours them [34] (R. Muscheler & Å. Wallin, pers. comm., 2023). This provides them with sufficient contrast in modern or Quaternary assemblages, but can render them more difficult to distinguish from indigenous grains of some deep-time assemblages that have undergone darkening via thermal maturation (e.g., [89–92]). If this is problematic, an alternative method of increasing contrast is to utilize fluorescence microscopy. Owing to the distinctive autofluorescence response of some exotic grains (e.g., *Lycopodium* spores; Fig 8), the contrast between markers and other grains (particularly in thermally matured assemblages) can be greatly enhanced, thus expediting accurate data collection.
3. ***Sensitive to heterogeneous spatial distribution:*** The FOVS method is susceptible to heterogeneity in specimen distribution across the study surface area. This stems from the primary difference between the linear and FOVS methods: the spatial variance of common specimen abundance during the calibration count phase. If the number of specimens per field of view is highly variable during these counts (i.e., high $s_{3}$), the sample’s total error ($\sigma_{\text{F}x}$) will increase correspondingly, resulting in decreased precision. To circumvent this issue, it is essential that the spatial ranges of both the calibration and extrapolation counts are near equivalent. In other words, the regions chosen for both the calibration- and extrapolation-count fields of view should overlap or be located close to each other, thus ensuring that the specimen density statistics (means and standard deviations) for both counts will be roughly equivalent (Fig 2A). In the case of organic microfossil slides, we recommend avoiding the slide margins where densities tend to be lower than the medial areas, since these may result in significant edge effects (Fig 2A). Our preliminary Monte Carlo computer experiments have shown that the dispersed nature of the FOVS method counting (as opposed to the contiguous counting in the linear method) may help to compensate for heterogeneity in specimen distribution by sampling a broader region.
  Furthermore, we have acknowledged that the simulated and microfossil datasets have different assumptions about particle distributions. The particles on microscope slides do not necessarily follow random (Poisson) distributions, and may be prone to specimen-specimen interactions, e.g., particle clumping or artificial dispersal. While a range of preparation techniques have been advanced to promote homogenous distributions for microorganisms (settling chambers or surfactants, e.g., [93,94]), this cannot be assured in all cases. Moreover, while such techniques may prevent clumping, they may result in non-Poisson homogeneity. Other particle distributions could be proposed, however we have not yet systematically assessed the effects of distribution heterogeneity and this should be the focus of future efforts.

To summarise, the FOVS method is designed for absolute abundance estimates of one or more targets with higher precision and/or reduced data collection effort than the linear method. However, it is not optimised for collecting relative abundances of diverse specimen categories. Moreover, to maximise the accuracy and precision of the FOVS method, we recommend: 1, high visible contrast between markers and other specimens; and 2, study area ranges for both calibration and extrapolation counts that are equivalent and homogeneous.

#### Which technique to use?

Regardless of the count type, the most precise estimates of concentration are achieved when the target specimens (e.g., terrestrial organic microfossils, $c_{t}$) and exotic markers (e.g., introduced *Lycopodium* spores) are close to equivalent. However, the FOVS method is particularly resilient to disproportionate ratios.

We have provided a test for determining the superior (highest precision and lowest effort) method for a given assemblage (Eqn 20). Highly precise estimates for the necessary parameters of this test—specifically, ω, $\hat{u}$ and ${\overset{―}{Y}}_{3x}$—will only be available after a substantial amount of data collection (e.g., after the calibration counts of the FOVS method; see ‘field-of-view subsampling (FOVS) method: operation’). However, sufficient estimates of two of the parameters (the target-to-marker ratio, $\hat{u}$, and the target density per field of view, ${\overset{―}{Y}}_{3x}$) will generally be obvious upon cursory inspection of an assemblage. The third parameter (transition-to-count ratio, ω) is the time variable, and is dependent on the target types, observation methods, and observer habits, which are largely consistent across assemblages in a study; this factor can be estimated even before inspecting a given assemblage. Hence, ‘ballpark’ values of these parameters will typically be easy to obtain; with these values, we recommend utilising Eqn 20 (Fig 1, steps 1, 2) upon first examination of each assemblage for determining the most efficient count method.

In most cases, the FOVS method is more efficient, except for those with extremely low specimen densities (very low ${\overset{―}{Y}}_{3x}$) and/or where markers and targets are near-equivalent ($\hat{u}\approx 1$). We recommend that in cases where target-to-marker ratios approach 1:1, the ‘linear method’ of counting should be preferred. This is because this approach also provides additional data that may be important to the researcher (e.g., relative population abundances), and does not require additional conditions such as high marker contrast, or consistent areas for both calibration and extrapolation counts. In all other cases, the FOVS method should provide superior concentration and precision estimates.

One specific application of the FOVS method we envision: the re-analysis of previously collected samples with suboptimal target-to-marker ratios. Our comparisons of the count methods (summarised in Table 2) show that suboptimal target-to-marker ratios will very likely result in unsatisfactory linear method precisions for most applications. However, samples with these suboptimal ratios may warrant reanalysis using the FOVS method (note: a prerequisite for these reanalyses is the inclusion of exotic markers during original sample preparation). Hence, the FOVS method enables a robust method for replicating past findings and preventing future ‘file-drawer effects’ (whereby null results are dismissed [95,96]). Lastly, we have observed from preliminary experiments with the Monte Carlo simulations that the relative efficiency of the methods is largely dependent on the homogeneity of the specimen distributions. We have not yet quantified these observations, but given that lab-based samples may be quite heterogeneous, we recommend that this important question should be investigated in future work.

The primary considerations in the choice of methods are summarised in Table 2. To aid in the determining the most appropriate count method, we have: 1, provided a step-by-step flowchart (Fig 1); and 2, integrated the relevant calculations into the user-friendly ‘absolute abundance calculator’ application we have provided (see instructions in the ‘software interface for calculating parameters of the linear and FOVS methods’ section). Moreover, we have also supplied the simulation Matlab code with which the reader may experiment to inform their own methodology (S2 File). See S2 Table for a full list of the relevant parameters.

## Conclusions

When describing the absolute abundance method first introduced in the 1960s (the ‘linear method’, herein), Maher [31] stated (p. 154): “considering the potential of the [absolute abundance] method, it is surprising it is not more widely used.” We echo this sentiment and aim to encourage the wider use of absolute abundance estimation by providing a new technique: the field-of-view subsampling (FOVS) method. In essence, the FOVS method requires only a few simple input parameters, in addition to those required for the well-established linear method: the standard deviation of specimens per field of view ($s_{3}$), and the numbers of fields of view counted ($N_{3C}$ and $N_{3E}$).

Through a combination of computer-simulated (Monte Carlo) and empirical (terrestrial organic microfossil) datasets, we have demonstrated that the FOVS method provides greater efficiency than the linear method under most conditions. The variables that had the greatest impacts on the relative efficiency of the methods were (in order of decreasing influence): 1, the ratio of targets to marker specimens; 2, the spatial density of targets; and 3, the relative duration of field-of-view translation vs specimen counting. The suitability of the FOVS method across a broader range of data sets stems from the potential of this approach to glean multiple, precise absolute abundance estimates even from assemblages that might not be optimised for absolute abundance counts. The FOVS method has the added benefit of demonstrating more accurate precision estimates than the linear method in almost all cases. The FOVS method may also provide greater precision and/or lower sampling effort (than the linear method) for samples that contain highly heterogeneous specimen distributions, but this remains to be tested. We hope that the versatility of the new count technique will not only encourage a broader adoption of absolute abundance estimation in the future, but facilitate the re-examination of legacy data sets (e.g., previously prepared samples spiked with suboptimal numbers of exotic markers). This study has provided a stepwise process for choosing the optimal method for each data set, aided by a user-friendly software interface for all key calculations and the source code for the computer simulations herein.

The FOVS method could, in principle, be readily applied to any count data that involves: 1, area-based sampling; and 2, readily identifiable markers of known quantity and predictable spatial distributions. Hence, while initially developed and applied to organic microfossil assemblages, we envision its application to other well-controlled, ‘petri-dish’ contexts, where targets specimens have been isolated from their original populations, e.g., blood, lake or sediment samples.

## Supporting information

S1 FigSimulated precisions of the linear and FOVS methods for different target-to-marker ratios ($\overset{―}{\overset{―}{u}}$).A, Total standard error scaled to standardised effort (${\tilde{\sigma }}_{M}$), expressed as %; see S15 and S16 Eqns. B, Percentage difference between exact total standard error (${\tilde{\sigma }}_{exact,M}$) and estimated total standard error (including the finite population correction; FPC), expressed as %; i.e., the values of S20 Eqn relative to S18 Eqn (linear method) or S21 Eqn relative to S19 Eqn (FOVS method). See S4–S14 Tables for the data expressed here.(EPS)

S1 TableList of statistical terms and their descriptions.Confidence interval functions follow Maher [31], as updated by Mertens et al. [49]. ^a^Note: In this paper we have used the factor 1/(N−1) (‘Bessel’s correction’, [97]) to give an unbiased estimator of the sample variances and sample standard deviations.(DOCX)

S2 Table Key input and output parameters for the absolute abundance calculations. Approximations for the ‘pre-collection’ outputs can all be achieved before completing data collection for a given sample (e.g., prior to, or during, the calibration counts of the FOVS method), and can guide a user’s choice of method and/or data collection parameters. ^a^Confidence interval functions provided by Maher [31] as updated by Mertens et al. [49], terms listed in S1 Table.
(DOCX)

S3 TableEmpirical data set of terrestrial organic microfossil data from Permian–Triassic assemblages of the Bonneys Plain-1 core, Tasmania Basin, southeastern Australia.Parameters for both of the count methods discussed in this study (linear and FOVS) are provided, including embedded formulae where relevant. Descriptions of formulae and terms follow those outlined in the manuscript or in S1 Table. Blue column headers indicate input parameters; CI = confidence interval; CL = confidence level; FOV = field of view; reg. = registration (samples registered at the Swedish Museum of Natural History); s.d. = standard deviation; t.u. = time units. ^a^Indicates values that are utilised when n<x (i.e., when u^<1).(XLSX)

S4 TableSimulation summary data (simulation conditions 1 of 11).Comparison table of terrestrial organic microfossil concentration estimates ($c_{M}$) from Eqn 1 (when M=L) or Eqn 4 (when M=F), and their associated errors and sampling efforts from a simulated data set of randomly distributed target and exotic specimens. Parameters: total targets in study area =30,000; total markers in study area =30,000; target-to-marker ratio = 1:1 (i.e., $\overset{―}{\overset{―}{u}}=1$); x count (linear method) =482; simulated iterations $=10^{6}$; ω=2; $N_{3C}=17$; $N_{3E}=17$; ${\overset{―}{Y}}_{3}=27$; ${\overset{―}{Y}}_{3}^{*}=\infty$. Since ${\overset{―}{Y}}_{3}<{\overset{―}{Y}}_{3}^{*}$, the linear method is more efficient for this assemblage.(DOCX)

S5 TableSimulation summary data (simulation conditions 2 of 11).Comparison table of terrestrial organic microfossil concentration estimates ($c_{M}$) from Eqn 1 (when M=L) or Eqn 4 (when M=F), and their associated errors and sampling efforts from a simulated data set of randomly distributed target and exotic specimens. Parameters: total targets in study area =30,000; total markers in study area =25,000; target-to-marker ratio = 6:5 (i.e., $\overset{―}{\overset{―}{u}}=1.2$); x count (linear method) =524; simulated iterations $=10^{6}$; ω=2; $N_{3C}=17$; $N_{3E}=20$; ${\overset{―}{Y}}_{3}=27$; ${\overset{―}{Y}}_{3}^{*}=132$. Since ${\overset{―}{Y}}_{3}<{\overset{―}{Y}}_{3}^{*}$, the linear method is more efficient for this assemblage.(DOCX)

S6 TableSimulation summary data (simulation conditions 3 of 11).Comparison table of terrestrial organic microfossil concentration estimates ($c_{M}$) from Eqn 1 (when M=L) or Eqn 4 (when M=F), and their associated errors and sampling efforts from a simulated data set of randomly distributed target and exotic specimens. Parameters: total targets in study area =30,000; total markers in study area =20,000; target-to-marker ratio = 3:2 (i.e., $\overset{―}{\overset{―}{u}}=1.5$); x count (linear method) =574; simulated iterations $=10^{6}$; ω=2; $N_{3C}=17$; $N_{3E}=25$; ${\overset{―}{Y}}_{3}=27$; ${\overset{―}{Y}}_{3}^{*}=29.95$. Since ${\overset{―}{Y}}_{3}<{\overset{―}{Y}}_{3}^{*}$, the linear method is more efficient for this assemblage.(DOCX)

S7 TableSimulation summary data (simulation conditions 4 of 11).Comparison table of terrestrial organic microfossil concentration estimates ($c_{M}$) from Eqn 1 (when M=L) or Eqn 4 (when M=F), and their associated errors and sampling efforts from a simulated data set of randomly distributed target and exotic specimens. Parameters: total targets in study area =30,000; total markers in study area =15,000; target-to-marker ratio = 2:1 (i.e., $\overset{―}{\overset{―}{u}}=2$); x count (linear method) =635; simulated iterations $=10^{6}$; ω=2; $N_{3C}=17$; $N_{3E}=33$; ${\overset{―}{Y}}_{3}=27$; ${\overset{―}{Y}}_{3}^{*}=11.87$. Since ${\overset{―}{Y}}_{3}>{\overset{―}{Y}}_{3}^{*}$, the FOVS method is more efficient for this assemblage.(DOCX)

S8 TableSimulation summary data (simulation conditions 5 of 11).Comparison table of terrestrial organic microfossil concentration estimates ($c_{M}$) from Eqn 1 (when M=L) or Eqn 4 (when M=F), and their associated errors and sampling efforts from a simulated data set of randomly distributed target and exotic specimens. Parameters: total targets in study area =30,000; total markers in study area =10,000; target-to-marker ratio = 3:1 (i.e., $\overset{―}{\overset{―}{u}}=3$); x count (linear method) =711; simulated iterations $=10^{6}$; ω=2; $N_{3C}=17$; $N_{3E}=47$; ${\overset{―}{Y}}_{3}=27$; ${\overset{―}{Y}}_{3}^{*}=5.687$. Since ${\overset{―}{Y}}_{3}>{\overset{―}{Y}}_{3}^{*}$, the FOVS method is more efficient for this assemblage.(DOCX)

S9 TableSimulation summary data (simulation conditions 6 of 11).Comparison table of terrestrial organic microfossil concentration estimates ($c_{M}$) from Eqn 1 (when M=L) or Eqn 4 (when M=F), and their associated errors and sampling efforts from a simulated data set of randomly distributed target and exotic specimens. Parameters: total targets in study area =30,000; total markers in study area =5,000; target-to-marker ratio = 6:1 (i.e., $\overset{―}{\overset{―}{u}}=6$); x count (linear method) =806; simulated iterations $=10^{6}$; ω=2; $N_{3C}=16$; $N_{3E}=83$; ${\overset{―}{Y}}_{3}=27$; ${\overset{―}{Y}}_{3}^{*}=2.693$. Since ${\overset{―}{Y}}_{3}>{\overset{―}{Y}}_{3}^{*}$, the FOVS method is more efficient for this assemblage.(DOCX)

S10 TableSimulation summary data (simulation conditions 7 of 11).Comparison table of terrestrial organic microfossil concentration estimates ($c_{M}$) from Eqn 1 (when M=L) or Eqn 4 (when M=F), and their associated errors and sampling efforts from a simulated data set of randomly distributed target and exotic specimens. Parameters: total targets in study area =30,000; total markers in study area =3,000; target-to-marker ratio = 10:1 (i.e., $\overset{―}{\overset{―}{u}}=10$); x count (linear method) =852; simulated iterations $=10^{6}$; ω=2; $N_{3C}=15$; $N_{3E}=119$; ${\overset{―}{Y}}_{3}=27$; ${\overset{―}{Y}}_{3}^{*}=1.803$. Since ${\overset{―}{Y}}_{3}>{\overset{―}{Y}}_{3}^{*}$, the FOVS method is more efficient for this assemblage.(DOCX)

S11 TableSimulation summary data (simulation conditions 8 of 11).Comparison table of terrestrial organic microfossil concentration estimates ($c_{M}$) from Eqn 1 (when M=L) or Eqn 4 (when M=F), and their associated errors and sampling efforts from a simulated data set of randomly distributed target and exotic specimens. Parameters: total targets in study area =30,000; total markers in study area =2,000; target-to-marker ratio = 15:1 (i.e., $\overset{―}{\overset{―}{u}}=15$); x count (linear method) =877; simulated iterations $=10^{6}$; ω=2; $N_{3C}=14$; $N_{3E}=154$; ${\overset{―}{Y}}_{3}=27$; ${\overset{―}{Y}}_{3}^{*}=1.363$. Since ${\overset{―}{Y}}_{3}>{\overset{―}{Y}}_{3}^{*}$, the FOVS method is more efficient for this assemblage.(DOCX)

S12 TableSimulation summary data (simulation conditions 9 of 11).Comparison table of terrestrial organic microfossil concentration estimates ($c_{M}$) from Eqn 1 (when M=L) or Eqn 4 (when M=F), and their associated errors and sampling efforts from a simulated data set of randomly distributed target and exotic specimens. Parameters: total targets in study area =30,000; total markers in study area =1,500; target-to-marker ratio = 20:1 (i.e., $\overset{―}{\overset{―}{u}}=20$); x count (linear method) =890; simulated iterations $=10^{6}$; ω=2; $N_{3C}=14$; $N_{3E}=180$; ${\overset{―}{Y}}_{3}=27$; ${\overset{―}{Y}}_{3}^{*}=1.132$. Since ${\overset{―}{Y}}_{3}>{\overset{―}{Y}}_{3}^{*}$, the FOVS method is more efficient for this assemblage.(DOCX)

S13 TableSimulation summary data (simulation conditions 10 of 11).Comparison table of terrestrial organic microfossil concentration estimates ($c_{M}$) from Eqn 1 (when M=L) or Eqn 4 (when M=F), and their associated errors and sampling efforts from a simulated data set of randomly distributed target and exotic specimens. Parameters: total targets in study area =30,000; total markers in study area =1,000; target-to-marker ratio = 30:1 (i.e., $\overset{―}{\overset{―}{u}}=30$); x count (linear method) =903; simulated iterations $=10^{6}$; ω=2; $N_{3C}=13$; $N_{3E}=219$; ${\overset{―}{Y}}_{3}=27$; ${\overset{―}{Y}}_{3}^{*}=0.8821$. Since ${\overset{―}{Y}}_{3}>{\overset{―}{Y}}_{3}^{*}$, the FOVS method is more efficient for this assemblage.(DOCX)

S14 TableSimulation summary data (simulation conditions 11 of 11).Comparison table of terrestrial organic microfossil concentration estimates ($c_{M}$) from Eqn 1 (when M=L) or Eqn 4 (when M=F), and their associated errors and sampling efforts from a simulated data set of randomly distributed target and exotic specimens. Parameters: total targets in study area =30,000; total markers in study area =500; target-to-marker ratio = 60:1 (i.e., $\overset{―}{\overset{―}{u}}=60$); x count (linear method) =917; simulated iterations $=10^{6}$; ω=2; $N_{3C}=11$; $N_{3E}=283$; ${\overset{―}{Y}}_{3}=27$; ${\overset{―}{Y}}_{3}^{*}=0.5888$. Since ${\overset{―}{Y}}_{3}>{\overset{―}{Y}}_{3}^{*}$, the FOVS method is more efficient for this assemblage.(DOCX)

S1 File Additional methods. This document includes all additional supporting equations (including functions for the FOVS method when markers are more common than targets), and details of the statistical corrections used.
(DOCX)

S2 File Matlab code instructions for simulations. The codes used to generate the simulation data in this paper have not been optimised, and have some components that are either not used or not fully implemented. However, in the interests of full transparency, we include the exact versions of the code that we used for our results here: https://github.com/Palaeomays/FOVS_vs_linear_methods.git.
(DOCX)

S3 File Absolute abundance calculator user guide. This file includes instructions for use, a walkthrough and a worked example. The abundance calculator is accessible here: https://github.com/Palaeomays/FOVS_vs_linear_methods.git.
(DOCX)
