# Supplementary material for: Field-of-view subsampling: A novel ‘exotic marker’ method for absolute abundances, validated by simulation and microfossil case studies
Source: PLoS One. 2025 May 6;20(5):e0320887. doi: 10.1371/journal.pone.0320887 (PMC12054932; doi:10.1371/journal.pone.0320887)
Supplement: S1 Table — Confidence interval functions follow Maher [31], as updated by Mertens et al. [49]. aNote: In this paper we have used the factor 1/(N−1) (‘Bessel’s correction’, [97]) to give an unbiased estimator of the sample variances and sample standard deviations. (DOCX) [file pone.0320887.s002.docx]

Supporting information table 1

| **Term** | **Description** | **Method(s)** |
| --- | --- | --- |
| PRIMARY INPUTS AND OUTPUTS FOR CONCENTATION ESTIMATES | | |
| $\boldsymbol{c}$  **(variants:**  $\boldsymbol{c}_{\boldsymbol{L}}$**,** $\boldsymbol{c}_{\boldsymbol{Fx}}$**,** $\boldsymbol{c}_{\boldsymbol{Fn}}$**)** | Concentration of target specimens per unit mass (or volume); see Eqns 1 or 4. Unless specified, this is derived by the ‘linear method’ (i.e., $c=c_{L}$). $c_{F}$ indicates concentrations calculated from the FOVS method; $c_{Fx}$ or $c_{Fn}$ are used when the target or marker specimens are the foci of the calibration, respectively. In the microfossil case study herein, $c$ is the concentration of organic microfossils per gram of dried sediment (grains/g). | Both |
| $\boldsymbol{c}_{\boldsymbol{t}}$ | Concentration of terrestrial organic microfossils per gram of dried sediment (grains/g). This is used when the total terrestrial microfossil count is designated the target population of a microfossil assemblage. | Both |
| $\boldsymbol{x}$  **(variant:** $\boldsymbol{x}$**)** | Number of counted target specimens in a sample, while the total number of targets in a study area is denoted $x$. For the FOVS method, the target specimens of the calibration counts are typically the most common specimen type. If the markers are more common, see S1 Text. | Both |
| $\boldsymbol{n}$  **(variant:** $\boldsymbol{n}$**)** | Number of counted exotic markers (e.g., *Lycopodium* spores) in a sample, while the total number of markers in a study area is denoted $n$. | Both |
| $\hat{\boldsymbol{u}}$  **(variant:** $\boldsymbol{u}$**)** | Target-to-marker ratio in a sample count. If all targets and markers in the population were counted, their ratio would provide the true population target-to-marker value in a study area ($u$, where $u=\frac{x}{n}$). However, this is impractical for routine work. So, samples of targets and markers provide estimates of$u$ based on the ratio ($\hat{u}$) in a given sample count ($\hat{u}=\frac{x}{n}$) for the linear method, and $\hat{u}=\overline{Y}_{3x}/\overline{Y}_{3n}$ for the FOVS method. | Both |
| $\boldsymbol{N}_{\boldsymbol{1}}$ | Number of doses of exotic marker specimens. In the microfossil case study herein, these doses are tablets of *Lycopodium* spores. The details of the *Lycopodium* tablets utilised in the microfossil case study were provided by Lund University (see ‘case study 2—organic microfossils: methods’). | Both |
| ${\overline{\boldsymbol{Y}}}_{\boldsymbol{1}}$ | Mean number of exotic markers for one dose (e.g., number of *Lycopodium* spores in one tablet). | Both |
| $\boldsymbol{s}_{\boldsymbol{1}}$ | Sample standard deviation^a^ for one dose of exotic markers (e.g., standard deviation of *Lycopodium* spores in one tablet). | Both |
| $\boldsymbol{s}_{\boldsymbol{1}\boldsymbol{P}}$ | Proportional sample standard deviation^a^ of the number of exotic markers per dose $\left( s_{1P}=\frac{s_{1}}{\overline{Y}_{1}} \right)$. | Both |
| $\boldsymbol{s}_{\boldsymbol{m}}$ | Standard deviation of exotic markers added to the sample  ($s_{m}=\sqrt{N_{1}}\times s_{1}$). | Both |
| $\boldsymbol{m}$ | Total number of exotic markers added to the sample. | Both |
| $\bar{\boldsymbol{m}}$ | Estimated number of exotic markers added to the sample $(\bar{m}=N_{1}\times\overline{Y}_{1}).$ | Both |
| $\boldsymbol{T}$ | Error contribution from the exotic marker doses, e.g., *Lycopodium* tablets $(T={s_{1P}}^{2}/N_{1}$). | Both |
| $\boldsymbol{N}_{\boldsymbol{2}}$ | Total number of samples combined for the concentration estimate. | Both |
| ${\overline{\boldsymbol{Y}}}_{\boldsymbol{2}}$ | Mean sample mass (or volume); for single samples, this is the specific sample mass (or volume). | Both |
| $\overline{\boldsymbol{V}}$ | Total mass (or volume) of samples ($\overline{V}=N_{2}\times\overline{Y}_{2}$). | Both |
| $\boldsymbol{s}_{\boldsymbol{2}}$ | Standard deviation of sample mass (or volume); for single samples, this can be approximated by the square root of the mass (or volume). Hence, if $N_{2}=1$, then $s_{2}=\sqrt{\overline{Y}_{2}}$. | Both |
| $\boldsymbol{s}_{\boldsymbol{V}}$ | Standard deviation of mass (or volume) in sample ($s_{V}=\sqrt{N_{2}}\times s_{2}$). | Both |
| $\boldsymbol{N}_{\mathbf{3}\boldsymbol{C}}$ | Number of fields of view counted during the calibration counts. | FOVS |
| $\boldsymbol{N}_{\mathbf{3}\boldsymbol{E}}$ | Number of fields of view counted during the extrapolation counts. | FOVS |
| ${\overline{\boldsymbol{Y}}}_{\boldsymbol{3}}$ | Mean specimens (typically the target specimens, $x$) in each field of view. This is a measure of specimen density for the total sample area. If used as an estimate of target specimens per field of view, then $\overline{Y}_{3}=\overline{Y}_{3x}$ (see Eqn 3); if used for markers, then $\overline{Y}_{3}=\overline{Y}_{3n}$(see S7 Eqn). | FOVS |
| $\boldsymbol{s}_{\boldsymbol{3}}$ | Sample standard deviation^a^ for the common specimens (typically $x$, although this might be substituted for $n$; see S9 Eqn) counted during the calibration counts. | FOVS |
| $\boldsymbol{c}_{\boldsymbol{4}}$ | Correction factor to achieve an unbiased estimator of the population standard deviation. This factor is particularly important for small sample sizes, where the bias on the sample standard deviation can result in major differences from the population standard deviation. (See $\hat{s}_{3}$below for the application of the $c_{4}$ factor.) | FOVS |
| ${\hat{\mathbf{s}}}_{\boldsymbol{3}}$ | Estimator of the population standard deviation^a^ for the common specimens (typically $x$, although this might be substituted for $n$ in cases where the latter is more common; see S9 Eqn) counted during the calibration counts. Following Gurland & Tripathi [1], this unbiased estimator is calculated:  $\hat{s}_{3}=\frac{s_{3}}{c_{4}\left( N_{3C} \right)}$ , where  $c_{4}\left( N_{3C} \right)=\sqrt{\frac{2}{N_{3C}-1}\times\frac{\Gamma\left( \frac{N_{3C}}{2} \right)}{\Gamma\left( \frac{N_{3C}-1}{2} \right)}}$ .  In the above formulation, $\Gamma$ is the ‘gamma function’ [2]. | FOVS |
| $\boldsymbol{s}_{\boldsymbol{3}\boldsymbol{P}}$ | Proportional sample standard deviation^a^ of the number of common specimens in the calibration counts $\left( s_{3P}=\frac{\hat{s}_{3}}{\overline{Y}_{3}} \right)$. | FOVS |
| ***ω*** | Field-of-view transition effort factor, equal to the mean transition time between fields of view divided by the mean count time for each specimen. | Both |
| $\hat{\boldsymbol{x}}$ | Extrapolated number of counted target specimens for the extrapolation counts $\left( \hat{x}=\overline{Y}_{3x}\times N_{3E} \right)$; see formula in Eqn 3. | FOVS |
| $\hat{\boldsymbol{n}}$ | Extrapolated number of counted marker specimens for the extrapolation counts $\left( \hat{n}=\overline{Y}_{3n}\times N_{3E} \right)$; see formula in S7 Eqn. | FOVS (variant) |
| $\boldsymbol{A}$ | The degree of effort for each target specimen $\left( \frac{\omega}{\overline{Y}_{3}}+1+\frac{1}{\hat{u}} \right)$; see formula in Eqn 9. | Linear |
| $\boldsymbol{N}_{\boldsymbol{3}\boldsymbol{C}}^{\boldsymbol{*}}$ | Optimal number of calibration-count fields of view; see formulae in Eqns 14 and 23 (or variant S13 Eqn, which we recommend if $n>x$). | FOVS |
| $\boldsymbol{N}_{\boldsymbol{3}\boldsymbol{E}}^{\boldsymbol{*}}$ | Optimal number of extrapolation-count fields of view; see formulae in Eqns 15 and 24 (or variant S14 Eqn, which we recommend if $n>x$). | FOVS |
| $\boldsymbol{\delta}^{\boldsymbol{*}}$ | The optimal field-of-view count ratio ($\delta^{*}=\frac{N_{3E}^{*}}{N_{3C}^{*}}$); see formula in Eqn 16 (or variant S10 Eqn, which we recommend if $n>x$). | FOVS |
| ${\overline{\boldsymbol{Y}}}_{\boldsymbol{3}}^{\boldsymbol{*}}$ | Critical value of field-of-view target density [$\overline{Y}_{3}$] whereby either the FOVS or linear method is the superior choice. This parameter is utilised for the ‘method determination test’. If the most common specimens are the targets, then $\overline{Y}_{3}^{*}= \overline{Y}_{3x}^{*}$ (see Eqn 20); if the most common specimens are markers, then $\overline{Y}_{3}^{*}= \overline{Y}_{3n}^{*}$(see variant S11 Eqn, which we recommend if $n>x$). | Both |
| $\boldsymbol{\sigma}_{\boldsymbol{L}}$ | Total standard error on the concentration (in %); see formulae in Eqns 2 and 10. | Linear |
| $\boldsymbol{\sigma}_{\boldsymbol{F}}$ | Total standard error on the concentration (in %). If the most common specimens are the targets, then $\sigma_{F}= \sigma_{\text{F}x}$ (see Eqns 5 and 11); if the most common specimens are markers, then $\sigma_{F}= \sigma_{\text{F}n}$(see S9 Eqn). | FOVS |
| $\bar{\boldsymbol{\sigma}}$ | User-defined desired level of error (in %); see formulae in Eqns 21 and 25. | Both |
| $\boldsymbol{e}_{\boldsymbol{L}}$ | Data collection effort in dimensionless units of work; see formulae in Eqns 6 and 9. | Linear |
| $\boldsymbol{e}_{\boldsymbol{F}}$ | Data collection effort in dimensionless units of work; see formula in Eqn 7. | FOVS |
| $\boldsymbol{e}_{\boldsymbol{L}}\boldsymbol{(}\bar{\boldsymbol{\sigma}}\boldsymbol{)}$ | Data collection effort for a user-defined error ($\bar{\sigma}$), as a function of effort; see formula in Eqn 21. | Linear |
| $\boldsymbol{e}_{\boldsymbol{F}}\boldsymbol{(}\bar{\boldsymbol{\sigma}}\boldsymbol{)}$ | Data collection effort for a user-defined error ($\bar{\sigma}$), as a function of effort; see formula in Eqn 25. | FOVS |
| CONFIDENCE INTERVAL FUNCTIONS | | |
| $\boldsymbol{\alpha}$ | ${=tan}^{-1} [(\bar{m}/s_{m})/(\bar{V}/s_{V})]$ | Linear |
| $\boldsymbol{\beta}$ | ${=sin}^{-1} [1/\sqrt{\left( \bar{m}/s_{m} \right)^{2}+\left( \bar{V}/s_{V} \right)^{2}} ]$ | Linear |
| ${\mathbf{(}\boldsymbol{m}\mathbf{/}\boldsymbol{V}\mathbf{)}}_{\mathbf{max}}$ | ${=[s}_{m}\times\tan\left( \alpha+\beta\right)] /s_{V}$ | Linear |
| ${\mathbf{(}\boldsymbol{m}\mathbf{/}\boldsymbol{V}\mathbf{)}}_{\mathbf{min}}$ | ${=[s}_{m}\times\tan\left( \alpha-\beta\right)] /s_{V}$ | Linear |
| ${\hat{\boldsymbol{u}}}_{\boldsymbol{max}}$ | $=\frac{\hat{u}+\left[ \frac{1}{(2n)} \right]+\sqrt{\left[ \frac{\hat{u}\left( 1+\hat{u} \right)}{n} \right]+\left[ \frac{1}{{4n}^{2}} \right]}}{1-\left( \frac{1}{n} \right)}$ | Linear |
| ${\hat{\boldsymbol{u}}}_{\boldsymbol{min}}$ | $=\frac{\hat{u}+\left[ \frac{1}{(2n)} \right]-\sqrt{\left[ \frac{\hat{u}\left( 1+\hat{u} \right)}{n} \right]+\left[ \frac{1}{{4n}^{2}} \right]}}{1-\left( \frac{1}{n} \right)}$ | Linear |
| $\log\hat{\boldsymbol{u}}$ | $=\log(x/n)$ | Linear |
| $\boldsymbol{s}_{\mathbf{log}\hat{\boldsymbol{u}}}$ | $=(\log\hat{u}_{\max}-\log\hat{u}_{\min})/2$ | Linear |
| $\bar{\log\boldsymbol{m}\mathbf{/}\boldsymbol{V}}$ | $=\log(\bar{m}/\bar{V})$ | Linear |
| $\boldsymbol{s}_{\log\mathbf{(}\boldsymbol{m}\mathbf{/}\boldsymbol{V}\mathbf{)}}$ | $=[log {(m/V)}_{\max}-\log{(m/V)}_{\min}]/2$ | Linear |
| **log *F*** | $=Z\sqrt{{(s_{\log\hat{u}})}^{2}+{(s_{\log(m/V)})}^{2}}$ | Linear |
| **Log limit** | $=(\log\hat{u}+\bar{\log m/V}) \pm\log F$ | Linear |
| ***Z*-score** | This value denotes the distance (in standard deviation units) of an observed value from the mean. Below are some example *Z*-scores for commonly used confidence levels.  90% confidence level: $Z \sim1.65$  95% confidence level: $Z \sim1.96$  99% confidence level: $Z \sim2.58$… | Linear |
| $\boldsymbol{CI}_{\boldsymbol{max}}$ | Confidence interval maximum, ${CI}_{max}=\frac{\hat{u}\times\bar{m}\times F}{\bar{V}}$ | Linear |
| $\boldsymbol{CI}_{\boldsymbol{min}}$ | Confidence interval minimum, ${CI}_{min}=\frac{\hat{u}\times\bar{m}}{\bar{V}\times F}$ | Linear |

References

1. Gurland J, Tripathi RC. A simple approximation for unbiased estimation of the standard deviation. The American Statistician. 1971;25(4):30–2.

2. Davis PJ. Leonhard Euler's integral: A historical profile of the gamma function. The American Mathematical Monthly. 1959;66(10):849–69. doi: 10.1080/00029890.1959.11989422.
