# Supplementary material for: Field-of-view subsampling: A novel ‘exotic marker’ method for absolute abundances, validated by simulation and microfossil case studies
Source: PLoS One. 2025 May 6;20(5):e0320887. doi: 10.1371/journal.pone.0320887 (PMC12054932; doi:10.1371/journal.pone.0320887)
Supplement: S2 Table — Approximations for the ‘pre-collection’ outputs can all be achieved before completing data collection for a given sample (e.g., prior to, or during, the calibration counts of the FOVS method), and can guide a user’s choice of method and/or data collection parameters. aConfidence interval functions provided by Maher [31] as updated by Mertens et al. [49], terms listed in S1 Table. (DOCX) [file pone.0320887.s003.docx]

Supporting information table 2

| **Function** | **Method** | **Input parameters** | **Output parameter** | **Equation** |
| --- | --- | --- | --- | --- |
| PRE-COLLECTION OUTPUTS | | | | |
| **1) Optimal field-of-view count ratio** | FOVS | $\hat{u}$, $\overline{Y}_{3}$, $\omega$ | $\delta^{*}$ | Eqn 16 (or S10) |
| **2) Method determination 1 (minimum specimen density per field of view for FOVS method superiority)** | Both | $\hat{u}$, $\overline{Y}_{3}$, $\omega$ | $\overline{Y}_{3}^{*}$ | Eqn 20 (or S11) |
| **3) Optimal number of calibration-count fields of view (for user-defined desired error)** | FOVS | $N_{1}$, $\overline{Y}_{1}$, $s_{1}$,$\hat{u}$, $\overline{Y}_{3}$, $\omega$, $\bar{\sigma}$ | $N_{3C}^{*}$ | Eqn 14 (or S13) |
| **4) Optimal number of extrapolation-count fields of view (for user-defined desired error)** | FOVS | $N_{1}$, $\overline{Y}_{1}$, $s_{1}$, $\hat{u}$, $\overline{Y}_{3}$, $\omega$, $\bar{\sigma}$ | $N_{3E}^{*}$ | Eqn 15 (or S14) |
| **5) Predicted data collection effort (for user-defined desired error)** | Linear | $N_{1}$, $\overline{Y}_{1}$, $s_{1}$, $\hat{u}$, $\overline{Y}_{3}$, $\omega$, $\bar{\sigma}$ | $e_{L}\left( \bar{\sigma} \right)$ | Eqn 21 |
| **6) Predicted data collection effort (for user-defined desired error)** | FOVS | $N_{1}$, $\overline{Y}_{1}$, $s_{1}$, $\hat{u}$, $\overline{Y}_{3}$, $\omega$, $\bar{\sigma}$ | $e_{F}\left( \bar{\sigma} \right)$ | Eqn 25 |
| POST-COLLECTION OUTPUTS | | | | |
| **7) Concentration** | Linear | $N_{1}$, $\overline{V}$, $\overline{Y}_{1}$, $x$, $n$ | $c_{L}$ | Eqn 1 |
| **8) Total error** | Linear | $N_{1}$, $\overline{Y}_{1}$, $x$, $n$, $s_{1}$ | $\sigma_{\text{L}}$ | Eqn 2 |
| **9) Confidence intervals** | Linear | $N_{1}$, $\overline{Y}_{1}$, $x$, $n$, $s_{1}$, $N_{2}$, $s_{2}$, $Z$ | ${CI}_{min}$, ${CI}_{max}$ | ^a^ |
| **10) Concentration** | FOVS | $N_{1}$, $\overline{V}$, $\overline{Y}_{1}$, $x$, $n$, $\overline{Y}_{3}$, $f$ | $c_{Fx}$ | Eqn 4 (or S8) |
| **11) Total error** | FOVS | $N_{1}$, $\overline{Y}_{1}$, $s_{1}$, $x$, $n$, $\overline{Y}_{3}$, $s_{3}$ | $\sigma_{Fx}$ | Eqn 5 (or S9) |
