# Supplementary material for: Field-of-view subsampling: A novel ‘exotic marker’ method for absolute abundances, validated by simulation and microfossil case studies
Source: PLoS One. 2025 May 6;20(5):e0320887. doi: 10.1371/journal.pone.0320887 (PMC12054932; doi:10.1371/journal.pone.0320887)
Supplement: S9 Table — Comparison table of terrestrial organic microfossil concentration estimates (cM) from Eqn 1 (when M=L) or Eqn 4 (when M=F), and their associated errors and sampling efforts from a simulated data set of randomly distributed target and exotic specimens. Parameters: total targets in study area =30,000; total markers in study area =5,000; target-to-marker ratio = 6:1 (i.e., u――=6); x count (linear method) =806; simulated iterations =106; ω=2; N3C=16; N3E=83; Y―3=27; Y―3*=2.693. Since Y―3>Y―3*, the FOVS method is more efficient for this assemblage. (DOCX) [file pone.0320887.s010.docx]

Supporting information table 9

| **Parameter estimates** | **Linear method**  ($M=L$) | **FOVS method**  ($M=F$) |
| --- | --- | --- |
| **1) Concentration (**$\boldsymbol{c}_{\boldsymbol{M}}$**; specimens/unit size), mean** | 30004 | 29995 |
| **2) Sampling effort (**$\boldsymbol{e}_{\boldsymbol{M}}$**; time units), mean** | 1000 | 1003 |
| **3) Estimated scaled standard error (**${\tilde{\boldsymbol{\sigma}}}_{\boldsymbol{M}}$**; %), mean (S15 and S16 Eqns)** | 9.331 | 7.114 |
| **4) Exact total standard error from true concentration (**${\tilde{\boldsymbol{\sigma}}}_{\boldsymbol{exact,M}}$**; %) (S18 and S19 Eqns)** | 9.454 | 6.949 |
| **5) Estimated scaled standard error, with finite population correction (**${\hat{\boldsymbol{\sigma}}}_{\boldsymbol{M}}$**; %), mean (S20 and S21 Eqns)** | 9.206 | 6.946 |
| **6) Difference between rows 4 and 5 (%)** | 2.632 | 0.03893 |
| **Preferred method?** | No | Yes |
