# Supplementary material for: Field-of-view subsampling: A novel ‘exotic marker’ method for absolute abundances, validated by simulation and microfossil case studies
Source: PLoS One. 2025 May 6;20(5):e0320887. doi: 10.1371/journal.pone.0320887 (PMC12054932; doi:10.1371/journal.pone.0320887)
Supplement: S1 File — This document includes all additional supporting equations (including functions for the FOVS method when markers are more common than targets), and details of the statistical corrections used. (DOCX) [file pone.0320887.s016.docx]

Supporting information file 1

Analysis of precision as a function of effort and the ratio of common and rare grains

The precision of both methods will improve with increased effort. In this section, we analyse this more closely to quantify how much the precision improves, and how $\hat{u}$—the ratio of target-to-marker specimens (usually, common-to-marker specimens)—affects this improvement.

Linear method

For the linear method, increasing effort ($e_{L}$) results in increased precision (i.e., decreased error, $\sigma_{L}$). In practice, this translates into an increased length of our observational ‘window’ on the study area, resulting in greater quantities of targets and markers. We can obtain an estimate of this improvement by calculating the rate of change of $\sigma_{L}$ for changes in $e_{L}$. Mathematically, this is expressed as the derivative of $\sigma_{L}$ with respect to $e_{L}$ as follows:

$$\begin{aligned} \frac{\partial}{\partial e_{L}}\sigma_{L}=-50\frac{A\left( 1+\hat{u} \right)}{{e_{L}}^{2}\sqrt{T+\left( 1+\hat{u} \right)\frac{A}{e_{L}}}} \overset{\to}{e_{L}\to\infty} -50\frac{A\left( 1+\hat{u} \right)}{{e_{L}}^{2}\sqrt{T}}.\#\left( S1 \right) \end{aligned}$$

You will notice that the output values of S1 Eqn will be negative (since $A$ and $\hat{u}$ are both positive), which is expected since the error should reduce with increasing effort. Specifically, linear method error decreases with the reciprocal of the square of the effort. We can also see that the improvement to the precision depends on the target-to-marker ratio ($\hat{u}$) as well as the density of the target specimens across the study area (via the $\omega/ \overline{Y}_{3x}$ term in $A$).

Lastly, we can use S1 Eqn to rederive a result by Regal & Cushing [1] mentioned in the main text; specifically, that the highest precision estimates of the concentration are achieved when $\hat{u}=1$ (i.e., $x=n$). In other words, the question we are asking is how the error changes as $\hat{u}$ changes, which is expressed through the derivative:

$$\begin{aligned} \frac{\partial}{\partial\hat{u}}\sigma_{L}=50\frac{\left( \omega/\overline{Y}_{3x} \right)+1-\hat{u}^{-2}}{e_{L}\sqrt{T+\left( 1+\hat{u} \right)\frac{A}{e_{L}}}}\geq0.\#\left( S2 \right) \end{aligned}$$

We see that the output values are always non-negative, since both $\omega$ and $\overline{Y}_{3x}$ are greater than zero, and $\hat{u}\geq1$. (Recall that in this formalism, we have assumed $x\geq n$.) So, this means that the error ($\sigma_{L}$) increases (=precision decreases) as the target-to-marker ratio ($\hat{u}$) increases, with $\hat{u}=1$ giving the minimum error, exactly as Regal and Cushing [1] claimed.

FOVS method

To validly compare the efficiencies of the FOVS and linear methods, we need to characterise the relationship between FOVS method error ($\sigma_{F}$) and effort ($e_{F}$). As noted in the main text, a first step is to determine the optimal ratio of calibration- and extrapolation-count fields of view, which will simplify the relationship to a single dimension. This was done by taking the derivative of the FOVS method error function (Eqn 5) and setting it equal to zero, resulting in Eqns 14 and 15. With these optimal values, we can then derive expressions analogous to S1 and S2 Eqns. These derivations are detailed below.

By differentiating Eqn 11 with respect to each of the two independent variables ($N_{3C}$ and $N_{3E}$), we can calculate the effect that the target-to-marker ratio ($\hat{u}$) and target specimen density ($\overline{Y}_{3x}$) have on FOVS method precision with the following two equations:

$$\begin{aligned} \frac{\partial}{\partial N_{3C}}\sigma_{F}\left( N_{3C},N_{3E} \right)=-50\frac{1}{{N_{3C}}^{2}\overline{Y}_{3x}\sqrt{T+\frac{1}{N_{3C}\overline{Y}_{3x}}+\frac{\hat{u}}{N_{3F}\overline{Y}_{3x}}}}\#\left( S3 \right) \end{aligned}$$

and

$$\begin{aligned} \frac{\partial}{\partial N_{3E}}\sigma_{F}\left( N_{3C},N_{3E} \right)=-50\frac{\hat{u}}{{N_{3E}}^{2}\overline{Y}_{3x}\sqrt{T+\frac{1}{N_{3C}\overline{Y}_{3x}}+\frac{\hat{u}}{N_{3E}\overline{Y}_{3x}}}} .\#\left( S4 \right) \end{aligned}$$

Since $N_{3E}$ and $N_{3C}$ determine the effort for the FOVS method, these equations suggest that the increase in precision decreases with the reciprocal of the square of the effort, akin to the linear method (S1 Eqn). This should follow because of the linear relationship between $N_{3C}$, $N_{3E}$ and $e_{F}$ in Eqn 7.

To more explicitly characterise how error changes for increased effort, we use Eqn 17 (which used the optimal numbers of calibration- and extrapolation-count fields of view to express error as a function of effort) and differentiate it with respect to $e_{F}$ to find

$$\begin{aligned} \frac{\partial}{\partial e_{F}}\sigma_{F}=-\frac{50\left( \left[ 1+\hat{u} \right]\omega+2\overline{Y}_{3x}+2\sqrt{\left[ \omega+\overline{Y}_{3x} \right]\left[ \omega\hat{u}+\overline{Y}_{3x} \right]} \right)}{e_{F}^{2}\overline{Y}_{3x}\sqrt{T+\frac{1}{e_{F}\overline{Y}_{3x}}\left( \left[ 1+\hat{u} \right]\omega+2\overline{Y}_{3x}+2\sqrt{\left[ \omega+\overline{Y}_{3x} \right]\left[ \omega\hat{u}+\overline{Y}_{3x} \right]} \right)}}\overset{\to}{e_{F}\to\infty}-\frac{50\left( \left[ 1+\hat{u} \right]\omega+2\overline{Y}_{3x}+2\sqrt{\left[ \omega+\overline{Y}_{3x} \right]\left[ \omega\hat{u}+\overline{Y}_{3x} \right]} \right)}{e_{F}^{2}\overline{Y}_{3x}\sqrt{T}}.\#\left( S5 \right) \end{aligned}$$

This shows that, similar to S1 Eqn, as we increase the amount of effort, the improvement in precision decreases as the reciprocal of the square of the effort. So, we can conclude that the FOVS method has similar asymptotic behaviour to the linear method, and so the FOVS method is no worse that the linear method.

Lastly, we can also find the value of $\hat{u}$ that minimises the error by calculating an expression analogous to S2 Eqn, which was formulated for the linear method. We differentiate S5 Eqn with respect to $\hat{u}$ to obtain

$$\begin{aligned} \frac{\partial}{\partial\hat{u}}\sigma_{F}=\frac{50\omega\left( \omega\hat{u}+\overline{Y}_{3x}+\sqrt{\left[ \omega+\overline{Y}_{3x} \right]\left[ \omega\hat{u}+\overline{Y}_{3x} \right]} \right)}{e_{F}\overline{Y}_{3x}\left( \omega\hat{u}+\overline{Y}_{3x} \right)\sqrt{T+\frac{1}{e_{F}\overline{Y}_{3x}}\left( \left[ 1+\hat{u} \right]\omega+2\overline{Y}_{3x}+2\sqrt{\left[ \omega+\overline{Y}_{3x} \right]\left[ \omega\hat{u}+\overline{Y}_{3x} \right]} \right)}}\geq0.\#\left( S6 \right) \end{aligned}$$

From this equation, we see that any increase in $\hat{u}$ will increase the error. Therefore, the maximum precision is obtained where $\hat{u}=1$, as it was for the linear method.

Field-of-view subsampling (FOVS) method variant: if markers ($\boldsymbol{n}$) are more common than targets ($\boldsymbol{x}$)

The ‘common’ and ‘rare’ specimen types may differ between assemblages, as a function of concentration and/or number of introduced exotic markers. As such, the type of specimen for the calibration counts may vary between assemblages, and—for the purposes of precise concentration estimates—the most common specimen group should be preferred. This is because a larger sample size should result in lower standard deviations for the calibration counts. However, for most applications of this method, the targets ($x$) would be preferred as the more common specimen type, since disproportionately high counts of markers ($n$) do not provide additional details on the population of interest for our research (e.g., the indigenous fossils of an assemblage, as in the empirical case study presented herein). Put another way: our time is better spent collecting target, rather than marker, data, since these provide additional information about the assemblage beyond the target concentrations (e.g., relative abundances of the targets to other populations, occurrence of biostratigraphic index taxa, fossil preservation quality, etc.).

When the markers are the subject of the calibration counts, then their extrapolated sample abundance ($\hat{n}$) will need to be estimated from their calibration-count mean ($\overline{Y}_{3n}$) multiplied by the total number of extrapolation-count fields of vi$\hat{n}$ew ($N_{3E}$). This is analogous to Eqn 3, but $\hat{n}$ is substituted for $\hat{x}$ so that

$$\begin{aligned} \hat{n}=\overline{Y}_{3n}\times N_{3E}.\#\left( S7 \right) \end{aligned}$$

In this variant of the method, whereby markers are the subjects of the calibration counts, the concentrations of organic microfossils ($c_{Fn}$) can be estimated with a modified version of Eqn 1. Specifically, we can substitute $n$ with $\hat{n}$, and use the total number of target specimens from the extrapolation counts ($x$) as follows:

$$\begin{aligned} c_{Fn}=\frac{x\times N_{1}\times\overline{Y}_{1}}{\hat{n}\times\overline{V}}\#\left( S8 \right) \end{aligned}$$

Similarly, the calculation of total error (Eqn 5) necessitates a slight modification when the markers are the subject of the calibration count:

$$\begin{aligned} \sigma_{\text{F}n}=100\times\sqrt{\left( \frac{s_{1P}}{\sqrt{N_{1}}} \right)^{2}+\left( \frac{\sqrt{x}}{x} \right)^{2}+\left( \frac{s_{3P}}{\sqrt{N_{3C}}} \right)^{2}}.\#\left( S9 \right) \end{aligned}$$

As discussed for Eqn 11, for the purposes of the mathematical analysis, we assume the Poisson approximation $\left( \frac{n}{\sqrt{n}} \right)^{2}$ for the sample standard deviation $\left( \frac{s_{3P}}{\sqrt{N_{3C}}} \right)^{2}$. Here, the number of markers is given by $n=\overline{Y}_{3n}N_{3C}$ and the number of targets is $x=\overline{Y}_{3x}N_{3E}$. Then, by the same reasoning as Eqn 8, the following approximations can be made: $x=n\times\hat{u}$ for the linear method, and $\overline{Y}_{3x}=\overline{Y}_{3n}\times\hat{u}$ for the FOVS method. So, in cases where markers are more common than targets, we substitute these values into several of the equations expressed in the manuscript. Of particular importance to discriminating between the FOVS vs linear method is Eqn 16, which becomes

$$\begin{aligned} \delta^{*}\left( Y_{3n} \right)=\frac{N_{3E}^{*}(e_{F})}{N_{3C}^{*}(e_{F})}=\frac{1}{\hat{u}}\sqrt{\frac{\omega+Y_{3n}}{\omega\hat{u}^{-1}+Y_{3n}}} .\#\left( S10 \right) \end{aligned}$$

Hence, the equation for $\overline{Y}_{3n}^{*}$ (modified from Eqn 20), which indicates the critical density of markers (not targets) per field of view, can be calculated

$$\begin{aligned} \overline{Y}_{3n}^{*}=2\omega\frac{\hat{u}^{2}+\sqrt{\hat{u}^{3}\left( 1+\hat{u}\left[ \hat{u}-1 \right] \right)}}{\hat{u}\left( \hat{u}+1 \right)\left( \hat{u}-1 \right)^{2}},\#\left( S11 \right) \end{aligned}$$

whereby, if $\overline{Y}_{3n}$ > $\overline{Y}_{3n}^{*}$, then the FOVS method should be used.

By having the marker specimens as the subject of the calibration counts, the two-dimensional problem of FOVS method error outlined in Eqn 11 becomes

$$\begin{aligned} \sigma_{F}\left( N_{3C},N_{3E} \right)=100\sqrt{T+\frac{1}{N_{3E}(\overline{Y}_{3n}\times\hat{u})}+\frac{1}{N_{3C}\overline{Y}_{3n}}.}\#\left( S12 \right) \end{aligned}$$

When the markers are more common than targets, the optimal counts of calibration fields of view ($N_{3C}^{*}$) and extrapolation count fields of view ($N_{3E}^{*})$ in the FOVS method are given by

$$\begin{aligned} N_{3C}^{*}\left( \bar{\sigma} \right)=\frac{1}{\left( \bar{\sigma}/100 \right)^{2}-T}\left( \frac{\sqrt{\left[ \overline{Y}_{3n}+\omega\right]}+\sqrt{\left[ \overline{Y}_{3n}+\frac{\omega}{\hat{u}} \right]}}{\overline{Y}_{3n}\sqrt{\left[ \overline{Y}_{3n}+\omega\right]}} \right)\#\left( S13 \right) \end{aligned}$$

and

$$\begin{aligned} N_{3E}^{*}\left( \bar{\sigma} \right)=\frac{\frac{1}{\hat{u}}}{\left( \bar{\sigma}/100 \right)^{2}-T}\left( \frac{\sqrt{\left[ \overline{Y}_{3n}+\omega\right]}+\sqrt{\left[ \overline{Y}_{3n}+\frac{\omega}{\hat{u}} \right]}}{\overline{Y}_{3n}\sqrt{\left[ \overline{Y}_{3n}+\frac{\omega}{\hat{u}} \right]}} \right).\#\left( S14 \right) \end{aligned}$$

Note: for this study, we assume that the targets are more common than the exotic markers, unless stated otherwise.

Effort standardisation for simulation precision estimates

The purpose of the simulation was to compare the linear and FOVS method precisions for an equivalent amount of effort. However, the estimated effort of each method is a non-trivial combination of various deterministic and random factors (Eqns 6 and 7). Hence, it was not possible to set up the simulation to produce identical collection effort estimates for both methods. However, with some trial-and-error, we found sets of parameters that yielded roughly equal efforts, which we used as the inputs for the simulation (see simulation output data in S4–S14 Tables).

But with unequal effort, the reliability of any comparison between precisions of the two methods will suffer. To correct for this, we used rescaled total error estimates for each method ($\tilde{\sigma}_{L}$ for the linear method; $\tilde{\sigma}_{F}$ for the FOVS method)

$$\begin{aligned} \tilde{\sigma}_{L}=\sigma_{L}\frac{e_{L}}{\overline{e}}\#\left( S15 \right) \end{aligned}$$

$$\begin{aligned} \tilde{\sigma}_{F}=\sigma_{F}\frac{e_{F}}{\overline{e}}\#\left( S16 \right) \end{aligned}$$

where $\overline{e}$ is the average effort $(e_{L}+e_{F})/2$. This rescaling will increase the error of the method that is associated with more work, while decreasing the error of the method associated with less work, making for a fairer comparison. Row 3 in each of the S4–S14 Tables contain these values, and they are plotted in S1 Fig.

Deviations from exact abundances

As discussed in ‘case study 1—computer simulations: methods’, we know the exact numbers of specimens in the simulated data sets in each virtual study area, hence: the exact concentration ($c_{exact}$). This allows us to compare the standard deviation of the concentration prediction of the two methods ($c_{L}$ and $c_{F}$) from this known true value ($c_{exact}$). Using the conventional sampling standard deviation, the equation for this is

$$\begin{aligned} \sigma_{exact,M}=100 \sqrt{\frac{1}{\left( N-1 \right)\times{c_{4}(N)}^{2}}\sum_{j=1}^{N} \left( \frac{c_{M}-c_{exact}}{c_{exact}} \right)^{2}},\#\left( S17 \right) \end{aligned}$$

where $N$ is the number of iterations of the simulation, the subscript $M$ denotes the chosen method for the calculation (linear [$L$] or FOVS [$F$] method), and $c_{4}(N)$ is the standard deviation bias correction (see S1 Table and ‘$c_{4}$ correction’ below). Since the error calculations Eqns 2 and 5 are empirical approximations of the quantity in S18 Eqn, we can use the simulations to gauge the accuracy of each method to predict the error.

To keep the comparison between the methods fair, we need to apply the effort standardisations as defined in S16 and S17 Eqns, giving us the following two equations:

$$\begin{aligned} \tilde{\sigma}_{exact,L}=\sigma_{exact,L}\frac{e_{L}}{\overline{e}}\#\left( S18 \right) \end{aligned}$$

$$\begin{aligned} \tilde{\sigma}_{exact,F}=\sigma_{exact,F}\frac{e_{F}}{\overline{e}}.\#\left( S19 \right) \end{aligned}$$

The results are contained on rows 4, 5 and 6 of S4–S14 Tables.

Statistical corrections for data sets

Finite population correction

Note that S17 Eqn is defined explicitly for the known finite number of target specimens on the virtual study area. As mentioned in the main text (see ‘case study 1—computer simulations: methods’), there are finite population effects that can become important when sampling too much area of any finite population (e.g., virtual study areas in our simulations, or microfossil slides). In particular, the standard deviations of samples from a finite population will be lower than those from an infinite population, and this discrepancy inflates as the sample size increases. In order to ensure a correct comparison between the exact (effort-scaled) errors in S19 and S20 Eqns and the predicted errors in Eqns 2 and 5, we also calculate finite-population versions of $\sigma_{L}$ and $\sigma_{F}$ (see [2], p. 83) in S21 and S22 Eqns, respectively:

$$\begin{aligned} \hat{\sigma}_{L}=\frac{{100 e}_{L}}{\overline{e}}\times\sqrt{\left( \frac{\sqrt{x}}{x} \right)^{2}\left( \frac{x-x}{x} \right)+\left( \frac{\sqrt{n}}{n} \right)^{2}\left( \frac{n-n}{n} \right)}\#\left( S20 \right) \end{aligned}$$

and

$$\begin{aligned} \hat{\sigma}_{F}=\frac{{100 e}_{F}}{\overline{e}}\times\sqrt{\left( \frac{s_{3P}}{\sqrt{N_{3}}} \right)^{2}\left( \frac{x-x}{x} \right)+\left( \frac{\sqrt{n}}{n} \right)^{2}\left( \frac{n-n}{n} \right)}.\#\left( S21 \right) \end{aligned}$$

Note that these equations have the expected behaviour: as $x$ and $n$ approach the extrapolation population counts ($x$ and $n$, respectively), the errors approach zero; while for small $x$ and $n$ counts, the errors approximate the infinite-population cases in Eqns 2 and 5.

Comparisons of the scaled precision estimates between the two methods are illustrated in S1 Fig. We further note the finite population correction would not be typically required for working with real-world microfossil data, if the proportion of the slide covered by the total field-of-view areas is small. Moreover, the number of fossils per slide is itself a random variable, which offsets the reduction in variance as the total field-of-view area increases.

$\boldsymbol{c}_{\mathbf{4}}$ correction

As mentioned in the main text, we have applied the $c_{4}$ correction to the standard deviation calculations of $s_{3P}$ in Eqn 5, and this was done for both the simulated and real fossil data. To calculate the correction, we use the expression in S1 Table.

The correction is also used in S17 Eqn; however, the $N$ in that equation refers to the number of iterations of the Monte Carlo simulation that are run to generate the data for averaging. Our simulations were run 1,000,000 times (i.e., $N={10}^{6}$); under these conditions, the biasing correction in S17 Eqn is $c_{4}\left( N \right)\approx0.99999975$, which we approximate with $c_{4}\left( N \right)=1$. (In the code of the simulation, we have explicitly included the approximation $c_{4}\left( N \right)=1$ for $N>341$to avoid numerical errors, as the calculation software used herein (Matlab) provided infinite answers for $N\geq344$.) The problem lies in the calculations of the gamma functions ($\Gamma$; see S1 Table), which are equivalent to factorial functions. These functions grow extremely fast as $N$ increases, and very quickly exceeds the calculation precision of standard computer packages. By carefully adjusting the precision (or using software dedicated to this process) the accuracy can be increased; however, to within the accuracy reported within this study, we did not need a better approximation for S18 Eqn.

Jensen’s inequality

When calculating the concentration mean across multiple iterations of the same sample, an additional statistical correction needed to be accounted for: Jensen's inequality [3] (ch. 8.3). This implies that $\mathbb{E}\left( 1/X \right)\geq1/\mathbb{E}\left( X \right)$, where $X$ is a random variable with a non-zero mean. In other words, the average of a reciprocal is always greater than or equal to the reciprocal of the average. Since the calculations of the concentrations include a random variable in the denominator ($n$) for both the linear (Eqn 1) and FOVS (Eqn 4) methods, Jensen's inequality means that if we take the average of the concentration estimate from each simulation, then we overestimate the true average concentration. If results from multiple samples are being averaged over, then Jensen's inequality will need to be accounted for, regardless of the type of data (simulated or empirical). Since all empirical (microfossil) data analysed herein are from single samples, this correction was not needed.

There are several ways to correct for this [4], the choice of which depends on the absolute values of counted specimens (e.g., if our sample happened to have no specimens counted in the sampling region, we would be dividing by zero). For the purposes of our simulations, since our parameters never resulted in zero exotic marker counts, we could use the direct method of calculating the reciprocal average $N_{3E}/n$ (i.e., the number of extrapolation-count fields of view, which was fixed for each iteration of the simulations, divided by the total number of markers counted) as an unbiased estimator, since this is exactly $1/\mathbb{E}\left( X \right)$. However, if the reader's experimental setup is such that zero exotic marker counts are possible, then other estimators of the reciprocal of binomial proportions may be of use; see [4].

References

1. Regal RR, Cushing EJ. Confidence intervals for absolute pollen counts. Biometrics. 1979;35(3):557–65.

2. Scheaffer RL, Mendenhall III W, Ott RL, Gerow K. Elementary Survey Sampling. 7^th^ ed. Boston: Brooks/Cole; 2012. 436 p.

3. Dekking FM, Kraaikamp C, Lopuhaä HP, Meester LE. A Modern Introduction to Probability and Statistics: Understanding Why and How. London: Springer-Verlag; 2005. 488 p.

4. Wei J, He P, Tong T. Estimating the reciprocal of a binomial proportion. International Statistical Review [Internet]. 2023:[16 p.].
