# Supplementary material for: Field-of-view subsampling: A novel ‘exotic marker’ method for absolute abundances, validated by simulation and microfossil case studies
Source: PLoS One. 2025 May 6;20(5):e0320887. doi: 10.1371/journal.pone.0320887 (PMC12054932; doi:10.1371/journal.pone.0320887)
Supplement: S2 File — The codes used to generate the simulation data in this paper have not been optimised, and have some components that are either not used or not fully implemented. However, in the interests of full transparency, we include the exact versions of the code that we used for our results here: https://github.com/Palaeomays/FOVS_vs_linear_methods.git. (DOCX) [file pone.0320887.s017.docx]

Supporting information file 2

**Matlab code for simulations—Quick Start Guide**

Get Matlab ready:

- Download the Matlab files to your working directory.
- Make a **\SimData\** subdirectory in your working directory.
- Note that Matlab must have the Statistics and Machine Learning Toolbox installed (which is required to perform the t-tests).
  - If you run the code without this Toolbox installed, then you will receive an error. There should be a link in the error message that will install the Toolbox for you (depending on your Matlab distribution and licence).

To generate the data for:

- For Tables S4–S14 and Fig S1, use the file **BigFossilSimsV3.m** by typing

**>> BigFossilSimsV3**

on the Matlab command line and hit Enter.

- For Fig 3, use the file **SimStatsChecker.m** by typing

**>> SimStatsChecker(30000,1000,700,15,100000,1)**

on the Matlab command line and hit Enter.

- For Fig 6, use the file **PrecWRTWorkV3.m** by typing

**>> PrecWRTWorkV3**

on the Matlab command line and hit Enter.

Note that some of these files will take many hours (even days) to run. If you wish to run shorter versions, change the “**its**” parameter (i.e., iterations) to a smaller number. You can generate new simulations with different parameters using the more detailed instructions below.

**Detailed descriptions of code parameters**

**Data for S4–S14 Tables and S1 Fig**

Code:

- **[Main] BigFossilSimsV3.m**
  - **[Dependent] MicrofossilSimV3.m**
  - **[Dependent] MicrofossilSim_iV3.m**
  - **[Dependent] FOVoptimiserV1.m**

Use:

Specify the following variables in **BigFossilSimsV3.m**:

- [Line 3] **its**: simulated iterations, i.e., the number of independent Monte Carlo instances to generate for each set of parameters.
- [Line 22] **params**: **[Mx, Mn, tab]**
- **Mx**: the total number of targets on each virtual study area ($x$).
- **Mn**: the total number of markers on each virtual study area ($n$).
- **tab**: value of the dose error used in Eqns 2 and 5 ($s_{1}$).
  - Note: Multiple rows of this variable can be specified to run multiple batches, via: **[(first batch parameters); (second batch parameters); ...]**
- [Line 27] **omega**: this is the field-of-view transition factor ($\omega$); default is $\omega=2$.
- [Line 33] **effort**: the fixed value of sampling effort that the program tries to achieve for each method ($e_{L}$ for the linear method, $e_{F}$ for the FOVS method).
  - Linear method: Eqn 9 is used to choose the number of targets to count.
  - FOVS method: Eqns 14 and 15 are used to choose the optimal number of calibration- and extrapolation-count fields of view ($N_{3C}^{*}$ and $N_{3E}^{*}$, respectively), via the code **FOVoptimiserV1.m**.

Notes:

- Ensure that there is a **\SimData\** subdirectory for the program in which to store the data files.
- The command line output will be saved in a file called **BigFossilSimsV3_Opt_TX_itsY.txt**, where
  - **X** is 10000 times the **tablet error** (to ensure an integer); and
  - **Y** is the value of **its**.

**Data for Fig 3**

Code:

- **[Main] SimStatsChecker.m**
  - **[Dependent] MicrofossilSim_iCheck.m**

Use:

Call the function **SimStatsChecker(Mx,Mn,tlim,fn,its,fopt)**, where the arguments are:

- **Mx**: The total number of targets on each virtual slide ($x$).
- **Mn**: The total number of markers on each virtual slide ($n$).
- **tlim**: (Linear method) the number of targets to count in the window. This specific value of this parameter is not for the output of this function. It only needs to be a value that will allow the simulation to progress. The number 700 is a good default value.
- **fn**: (FOVS method) the number of extrapolation-count fields of view in which to count markers ($N_{3E}$).
- **its**: Simulated iterations, i.e., the number of independent Monte Carlo instances to generate for each set of parameters.
- **fopt**: Not used. Set to 1.

**Data for Fig 6**

Code:

- **[Main] PrecWRTWorkV3.m**
  - **[Dependent] WorkSimV3.m**
  - **[Dependent] WorkSimV3_i.m**

Use:

Specify the following variables in **PrecWRTWorkV3.m**

- [Line 7] **its**: Simulated iterations, i.e., the number of independent Monte Carlo instances to generate for each set of parameters.
- [Line 8] **bigfx**: The number of calibration counts for the "high calibration counts" sequence in Fig 6 (black plus).
- [Line 9] **medfx**: The number of calibration counts for the "medium calibration counts" sequence in Fig 6 (blue stars).
- [Line 10] **smallfx**: The number of calibration counts for the "low calibration counts" sequence in Fig 6 (red stars).
- [Lines 19–24] **params**: **[Mx, Mx, tlim, fnmax, omega]**
  - **Mx**: The total number of targets on each virtual slide ($x$).
  - **Mn**: The total number of markers on each virtual slide ($n$).
  - **tlim**: (Linear method) the number of targets to count in the window.
  - **fnmax**: Not used. Set to 1.
  - **omega**: This is the field-of-view transition factor ($\omega$); default is $\omega=2$.
  - Note: Multiple rows of this variable can be specified to run multiple batches, via: **[(first batch parameters); (second batch parameters); ...]**

The simulations currently assume that the marker dose (e.g., tablet of *Lycopodium* spores) error is zero, i.e.: $\left( \frac{s_{1P}}{\sqrt{N_{1}}} \right)^{2}=0$. If you wish to increase this, then change the following variable:

- [**WorkSimV3_i.m**, Line 33] **tab**: Value of the marker dose error used in Eqns 2 and 5 ($s_{1}$).

Notes:

- Ensure that there is a **\SimData\** subdirectory for the program to store the data files in.
- The command line output will be saved in a file called **WorkSimOpt_tab0_itsY.txt**, where:
  - **Y** is the value of **its**.

**tab0** records that the marker dose error is zero for the simulations. This is hard-coded and will not update if the value of tab is changed in **WorkSimV3_i.m**.
