# Supplementary material for: Field-of-view subsampling: A novel ‘exotic marker’ method for absolute abundances, validated by simulation and microfossil case studies
Source: PLoS One. 2025 May 6;20(5):e0320887. doi: 10.1371/journal.pone.0320887 (PMC12054932; doi:10.1371/journal.pone.0320887)
Supplement: S3 File — This file includes instructions for use, a walkthrough and a worked example. The abundance calculator is accessible here: https://github.com/Palaeomays/FOVS_vs_linear_methods.git. (DOCX) [file pone.0320887.s018.docx]

Supporting information file 3

Absolute abundance calculator user guide

The following document is a set of illustrated instructions for accessing and utilizing the ‘absolute abundance calculator’. This calculator was designed as an interface for helping users estimate a range of absolute abundance parameters when using exotic markers.

The calculator was published in the journal *PLoS One* as part of the study “Field-of-view subsampling: A novel ‘exotic marker’ method for absolute abundances, validated by simulation and microfossil case studies” (authors: Mays, C., Amores, M. and Mays, A.); DOI: [10.1371/journal.pone.0320887](https://doi.org/10.1371/journal.pone.0320887).

Accessing the calculator

The latest version of the calculator can be downloaded from this link: <https://github.com/Palaeomays/FOVS_vs_linear_methods.git>.

(Note: the following instructions and example corresponds to v.1.1.1 of the absolute abundance calculator; use the link above for instructions on the latest version.)

The calculator has an Open XML standard file format (extension ‘.xlsm’). Upon opening the file, you may see the following message, or similar: "Microsoft has blocked macros from running because the source of this file is untrusted". If so, see the instructions here: <https://github.com/Palaeomays/FOVS_vs_linear_methods/blob/main/README.md>)

Navigating the calculator

The calculator consists of a series of ‘windows’, each with buttons and fields (for data inputs or outputs). Some common buttons and/or windows include:

- Glossary: This button appears on each window of the calculator. When clicked, a new window appears comprising a list of abbreviated statistical terms in alphabetical order and their expansions. We recommend that new users open this window and move it to one side of their screens for reference.

(Note: users can also hover their mouse cursor over the abbreviated terms to see expanded versions of each.)

- Counting assistant: This button opens a window designed to serve as a digital tally sheet for simultaneously counting up to 10 specimen types (e.g., one marker and 9 targets) from a single sample. Upon saving (with the ‘next FOV’ or ‘end and export to spreadsheet’ buttons), data can be exported into two distinct datasheets within the same calculator file. These datasheets are labelled: 1, ‘Counting (Exhaustive)’, in which the data for each field of view are stored separately; and 2, ‘Counting (Summary)’, which includes sample statistics including the mean specimens per field of view (${\overline{\boldsymbol{Y}}}_{\boldsymbol{3}}$) and sample standard deviation ($\boldsymbol{s}_{\boldsymbol{3}}$). For additional instructions on how to use the counting assistant, read the pop-up window when the ‘counting assistant’ button is pressed.
- Clear inputs: This button enables a user to delete all inserted data. On the linear and FOVS data collection windows (see Figs 2 & 4, respectively), two variants of this button are provided: ‘clear input data’ (which deletes only the values in the input fields) and ‘clear all data’ (which deletes all input and output values). Upon pressing these buttons, users are required to confirm their choice before the values are deleted.
- Calculate: This button results in the calculation of the output parameters on the relevant window, based on the available inputs. If there are missing inputs, users will be prompted to provide these. Output parameters will be shown based on the available inputs, even if not all inputs are inserted.
- Start timer: This button initiates a timer, which can be used for estimating total count time (CT) and/or field-of-view transition time (TT). The timer can be paused at any time: upon pressing, the ‘start timer’ button changes to a ‘pause timer’ button.
- Export data to spreadsheet: This button appears on the linear and FOVS data collection windows. Upon pressing, a spreadsheet tab in the same file will open, which includes all of the input and output variables, with separate tabs generated for each of the two methods. If this button is pressed more than once within the data collection of a single method (linear or FOVS), then separate rows will be generated for each export.

Walkthrough with worked example

Upon opening the calculator file, you will be presented with the opening window.

1. Click on the ‘absolute abundance calculator’ button; this opens the ‘method determination test’ window.

**Method determination test window**

The calculations in this window utilize the equations of the ‘choosing the superior count method’ section of the published article. These assist users in applying the most efficient count method (linear or FOVS) for each specific sample.

1. Insert the required inputs (see example in Fig. 1).
2. Press the ‘calculate’ button. The calculator will then calculate the most efficient count method (linear or FOVS), based on the input sample parameters. This method will be displayed in the lowermost window, and the button of the recommended method will turn green.

(Note: users can skip this step by clicking directly on the corresponding button for their method of choice: ‘linear’ or ‘FOVS’.)


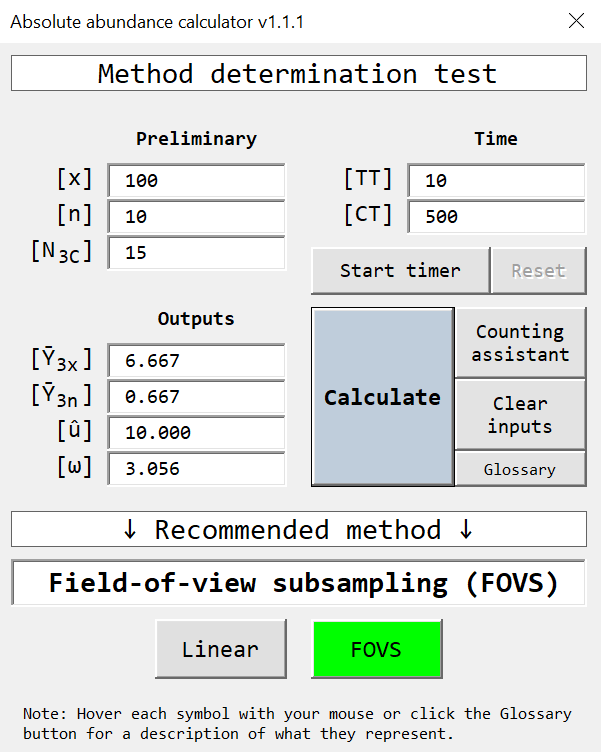


Figure 1. During preliminary data collection of this sample, 100 targets ($x$) and 10 markers ($n$) were counted across 15 fields of view ($N_{3C}$). The total count time (CT) was 500 seconds, and the transition time between fields of view (TT) was 10 seconds. From these inputs, the target-to-marker ratio ($\hat{u}$) was 10, the mean number of targets ($\overline{Y}_{3x}$) and markers ($\overline{Y}_{3n}$) per field of view were 6.667 and 0.667, respectively, and the field-of-view transition factor (*ω*) was 3.056. After pressing ‘calculate’, the FOVS method was indicated as the more efficient count method for this sample.

**Linear method data collection**

If the ‘linear’ button is selected on the ‘method determination test’ window, users will be taken to the ‘linear method data collection’ window. This is the primary data entry window for the linear method. It consists of the primary parameters on the main window, and sub-windows for the ‘marker and sample characteristics’ and ‘optional: optimisation data’. These sub-windows can be opened by pressing the associated buttons. If space on the desktop allows, users can even open these sub-windows and drag them next to the main window to simultaneously display all windows.

1. If the user had inserted values into the previous ‘method determination test’ window, the related input and output values will be displayed in their relevant places here.
2. Insert any additional required inputs on the main window and the ‘marker and sample characteristics’ sub-window (see example in Fig. 2). This should include a unique identifier for this sample in the ‘sample name’ field.

(Note: users need not insert values into the ‘optional: optimisation data’ sub-window).

1. Press ‘save’ on each sub-window after inserting the relevant data. If all data have been inserted and saved, the sub-window button will change from red to green.
2. Press calculate.


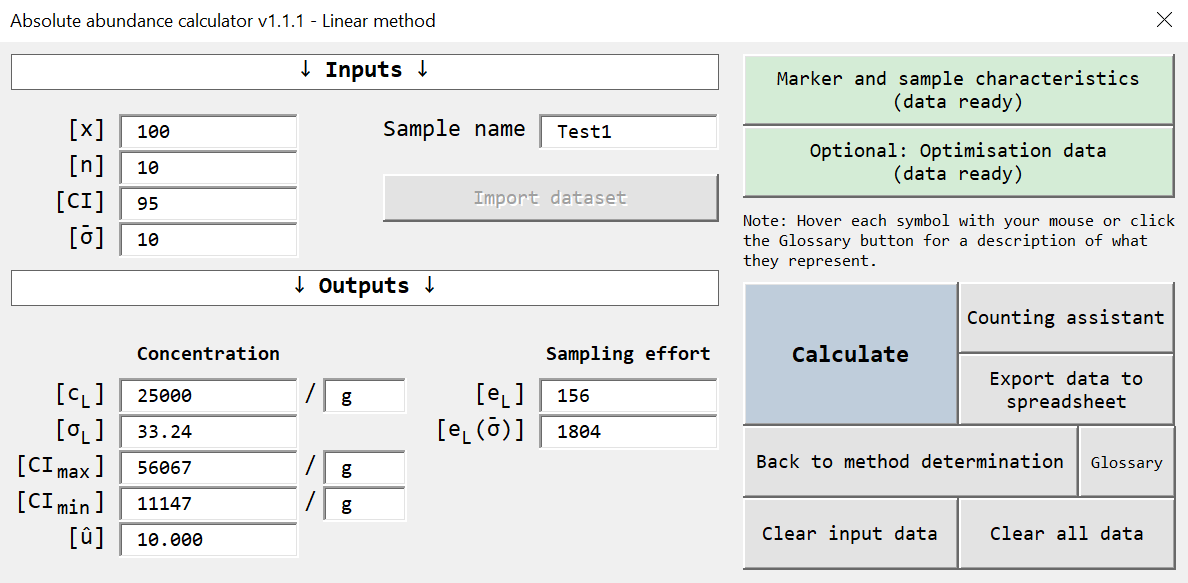

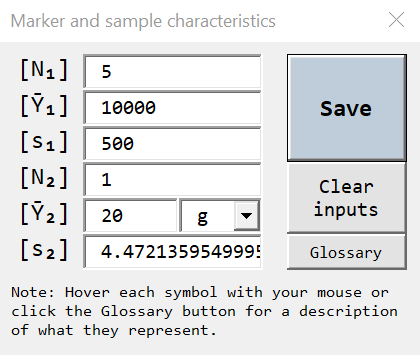

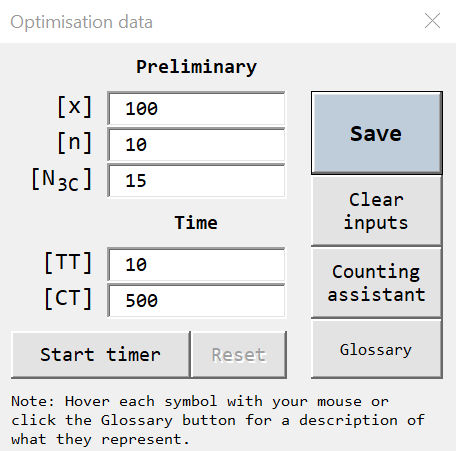


Figure 2. In this example, the sample name ‘Test1’, and a desired confidence interval range ($CI$) of 95% and desired error rate ($\bar{\sigma}$) of 10% were inserted into the main window (top). The following values pertaining to the exotic markers were entered into the ‘marker and sample characteristics’ sub-window: five doses of exotic markers ($N_{1}$) were added prior during sample processing, each of which comprised a mean ($\overline{Y}_{1}$) of 10,000 *Lycopodium* spores, with a standard deviation ($s_{1}$) of 500. On the same sub-window, the following sample characteristics were entered: the number of samples ($N_{2}$) was set to one, and the mass of this sample ($\overline{Y}_{2}$) was 20 g. Given that only one sample was included here, the sample standard deviation ($s_{2}$) was automatically approximated by the square root of the mass (see S1 Table). No additional optimisation data were needed, since these were prefilled from the previous ‘method determination test’ window inputs. With this combination of input data, the following outputs relevant to the target concentration were calculated: a concentration ($c_{L}$) of 25,000 specimens per gram with a total standard error ($\sigma_{L}$) of 33.24%, and a confidence interval minimum (${CI}_{min}$) and maximum (${CI}_{max}$) of 11,147 and 56,067 specimens per gram, respectively. The total sample collection effort ($e_{L}$) for the present data set was calculated as 156, but an estimated total effort value ($e_{L}(\bar{\sigma})$) of 1804 is needed to achieve the desired total error value ($\bar{\sigma}$) of 10%. In other words, more than an order of magnitude of additional data effort is required to achieve the desired precision.

Preliminary data window (FOVS method only)

Preliminary data will enable the calculation of the optimal amount of data collection for the FOVS method. If the FOVS method is selected on the ‘method determination test’ window, users will be asked whether they would like to insert these preliminary data.

1. If the user selects ‘yes’, the optional ‘preliminary data’ window will appear

(Note: this step can be skipped by pressing ‘no’.)

1. If the user had inserted values into the previous ‘method determination test’ window, these values will be displayed in the ‘preliminary’ and/or ‘time’ columns on this window.
2. Insert any additional required inputs on this window (see example in Fig. 3).

(Note: these data can be inserted at a later point. So, users can skip this window at any time by pressing the ‘skip to FOVS data collection’ button.)

1. Once all input variables have been inserted, press ‘calculate’.


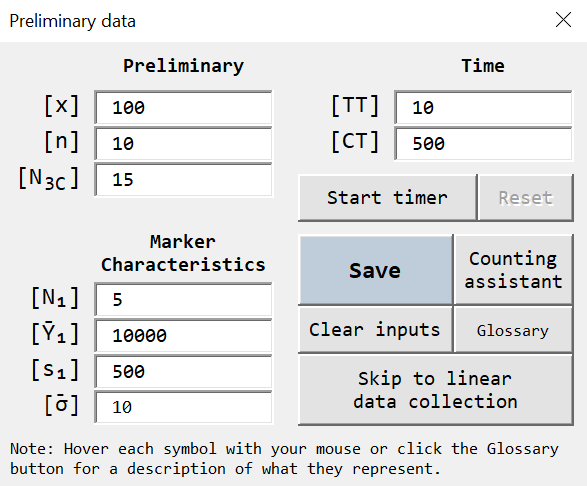


Figure 3. In addition to the previously inserted values from the method determination test (those in the ‘preliminary’ and ‘time’ columns; see Fig. 1), the marker characteristics were inserted. In this sample, five doses of exotic markers ($N_{1}$) were added prior during sample processing, each of which comprised a mean ($\overline{Y}_{1}$) of 10,000 *Lycopodium* spores, with a standard deviation ($s_{1}$) of 500. The desired error rate for the data collection ($\bar{\sigma}$) was set at 10%.

**FOVS method data collection**

This is the primary data entry window for the FOVS method. It consists of the primary parameters on the main window, and three sub-windows: 1, ‘marker and sample characteristics’; 2, ‘optional: optimisation data’; and 3, ‘field of view (FOV) calibration count’. This latter window is unique to the FOVS method window. Similar to the linear method, the sub-windows can be opened by pressing the associated buttons. If space on the desktop allows, users can open all sub-windows and drag them next the main window to simultaneously display all input and output data.

1. If the user had inserted values into one or both of the previous windows (‘method determination test’ and/or ‘preliminary data’), the related input and output values will be displayed in their relevant places.
2. Insert a unique identifier for this sample in the ‘sample name’ field of the main window.
3. Insert any additional required inputs in the ‘marker and sample characteristics’ sub-window.

(Note: users need not insert values into the ‘optional: optimisation data’ sub-window).

1. Insert all values in the ‘field of view (FOV) calibration count’ sub-window, including the sample standard deviation for the common specimens ($\boldsymbol{s}_{\boldsymbol{3}}$); typically, the common specimens are the target specimens*. If the ‘counting assistant’ has been used for the calibration count, the $\boldsymbol{s}_{\boldsymbol{3}}$ value is displayed automatically on the ‘Counting (Summary)’ tab.

(*Note: the targets [$\boldsymbol{x}$] are automatically set as the default subjects of the calibration counts. If, however, the saved data inputs parameters indicate that the markers [$\boldsymbol{n}$] are more common than the targets, a pop-up window will appear suggesting that the user change the focus of the calibration counts to the markers. Pressing this will enable that the most efficient data collection subject. See S1 File for more details.)

1. Press ‘save’ on each sub-window after inserting the relevant data. If all data have been inserted and saved, the sub-window button will change from red to green.
2. Insert the remaining input data on the FOVS data collection main window, including the markers (n) and fields of view ($\boldsymbol{N}_{\mathbf{3}\boldsymbol{E}}$) from the extrapolation count.
3. Press calculate.


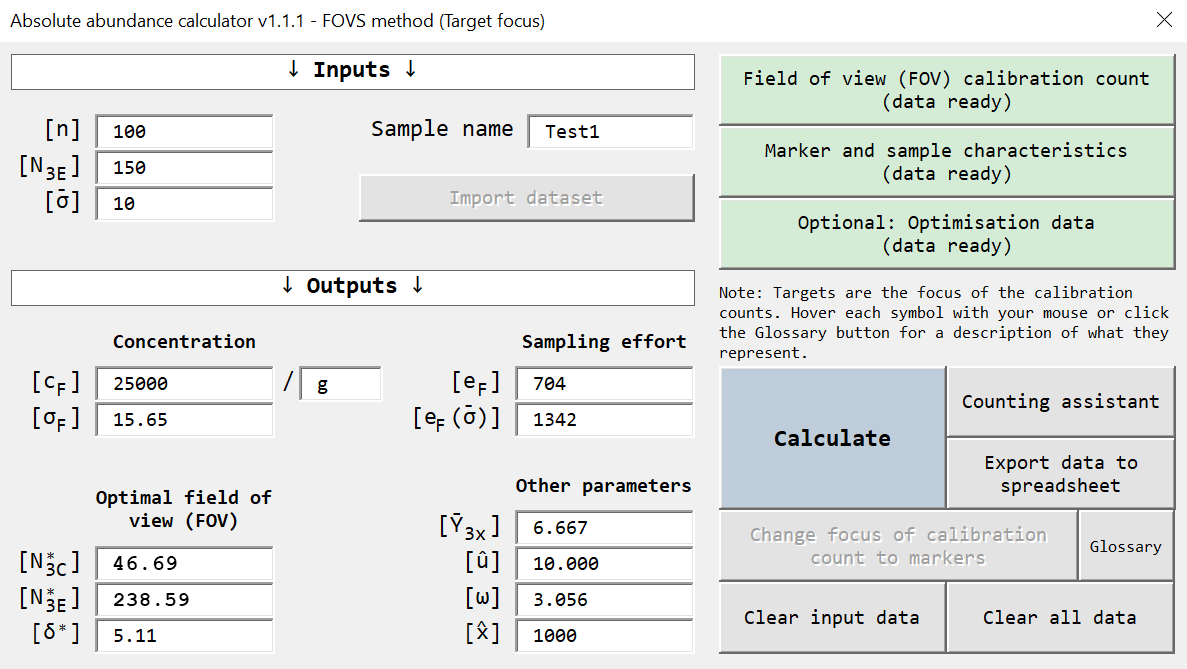


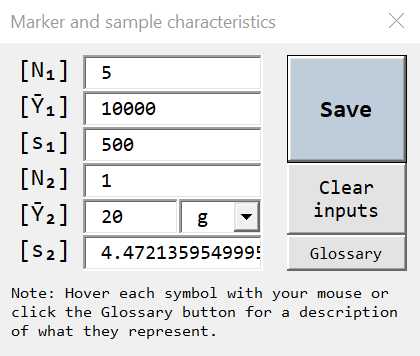

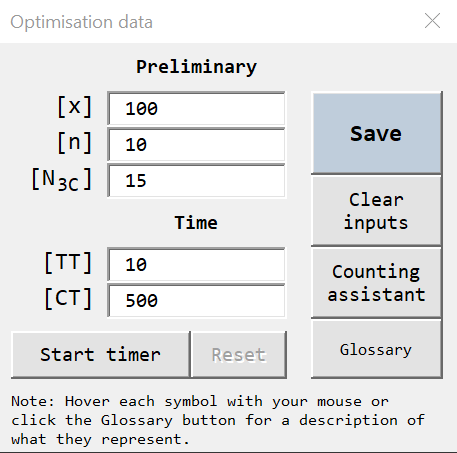


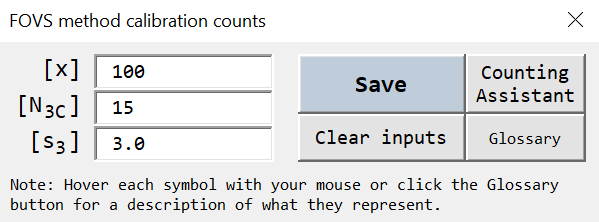


Figure 4. In our worked example, the same sample (Test1) was analyzed using both the linear and FOVS methods. Therefore, many of the inputs for the FOVS data collection (above) are the same as those used in Fig. 2. These include all the values for the ‘sample and marker characteristics’ and ‘optimisation data’ sub-windows, many of which were prefilled from the values previous entered on the ‘method determination test’ and ‘preliminary data’ windows. However, all inputs for the unique ‘FOVS method calibration counts’ sub-window (bottom) are required for the FOVS method. These calibration counts were conducted using the ‘counting assistant’; this provided the sample standard deviation for the target specimens ($s_{3}$) of 3.0 from a total number of target grains ($x$) of 100 across a series of 15 calibration count fields of view ($N_{3C}$). (Note: the latter two values were prefilled from the ‘preliminary data’ window). Following the calibration counts, extrapolation counts yielded 100 *Lycopodium* spore markers ($n$) across 150 fields of view ($N_{3E}$). Since it was the same sample analyzed as the linear method (Test1), many of the output parameters are the same, including the target-to-marker ratio ($\hat{u}$) of 10, mean number of targets ($\overline{Y}_{3x}$) per field of view of 6.667, the field-of-view transition factor (*ω*) of 3.056 and the concentration ($c_{F}$) of 25,000 target specimens per gram. Outputs unique to the FOVS method, however, include the optimal field-of-view ratio ($\delta^{*}$) of 5.11, which suggests that the user should collect data from approximately five times the number of extrapolation fields of view than calibration fields of view. Specifically, the optimal number calibration fields of view ($N_{3C}^{*}$) is c. 47, while the optimal number of extrapolation fields of view ($N_{3E}^{*}$) is c. 239. The precision of the present FOVS data is reflected by the total standard error ($\sigma_{F}$) of 15.65%, as a result of a data collection effort ($e_{F}$) of 704. To achieve the desired error rate ($\bar{\sigma}$) of 10%, a total effort value ($e_{F}(\bar{\sigma})$) of 1342 is estimated; this is substantially lower than the predicted effort value for the same precision using the linear method ($e_{L}(\bar{\sigma})$ = 1804; Fig. 2). However, to expend the lowest possible collection effort/time, it is important to aim for the optimal calibration and extrapolation FOV counts. These optimal count sizes can be estimated with preliminary counts for each new sample (Fig. 3).
